# Transcriptional profiles predict treatment outcome in patients with tuberculosis and diabetes at diagnosis and at two weeks after initiation of anti-tuberculosis treatment

**DOI:** 10.1016/j.ebiom.2022.104173

**Published:** 2022-07-15

**Authors:** Cassandra L.R. van Doorn, Clare Eckold, Katharina Ronacher, Rovina Ruslami, Suzanne van Veen, Ji-Sook Lee, Vinod Kumar, Sarah Kerry-Barnard, Stephanus T. Malherbe, Léanie Kleynhans, Kim Stanley, Philip C. Hill, Simone A. Joosten, Reinout van Crevel, Cisca Wijmenga, Julia A. Critchley, Gerhard Walzl, Bachti Alisjahbana, Mariëlle C. Haks, Hazel M. Dockrell, Tom H.M. Ottenhoff, Eleonora Vianello, Jacqueline M. Cliff, TANDEM Consortium

**Affiliations:** a Department of Infectious Diseases, Leiden University Medical Center, Leiden, the Netherlands; b Dept of Infection Biology and TB Centre, London School of Hygiene & Tropical Medicine, London, WC1E 7HT, United Kingdom; c SA MRC Centre for TB Research, DST/NRF Centre of Excellence for Biomedical Tuberculosis Research, Division of Molecular Biology and Human Genetics, Faculty of Medicine and Health Sciences, Department of Biomedical Sciences, Stellenbosch University, Cape Town, South Africa; d Mater Research Institute – The University of Queensland, Translational Research Institute, Brisbane, QLD, Australia; e TB-HIV Research Center, Faculty of Medicine, Universitas Padjadjaran, Bandung, Indonesia; f Hasan Sadikin General Hospital, Bandung, Indonesia; g University of Groningen, University Medical Center Groningen, Department of Genetics, Groningen, the Netherlands; h Department of Internal Medicine and Radboud Center for Infectious Diseases, Radboud University Medical Center, Nijmegen, the Netherlands; i Population Health Research Institute, St George's Hospital Medical School, University of London; j Centre for International Health, Division of Health Sciences, University of Otago, Dunedin, New Zealand; k Centre for Tropical Medicine and Global Health, Nuffield Department of Medicine, University of Oxford, Oxford, United Kingdom; l Department of Life Sciences, Brunel University London, United Kingdom

**Keywords:** Biomarkers, Tuberculosis, Treatment outcome, Diabetes mellitus

## Abstract

**Background:**

Globally, the tuberculosis (TB) treatment success rate is approximately 85%, with treatment failure, relapse and death occurring in a significant proportion of pulmonary TB patients. Treatment success is lower among people with diabetes mellitus (DM). Predicting treatment outcome early after diagnosis, especially in TB-DM patients, would allow early treatment adaptation for individuals and may improve global TB control.

**Methods:**

Samples were collected in a longitudinal cohort study of adult TB patients from South Africa (*n*  =  94) and Indonesia (*n*  =  81), who had concomitant DM (*n*  =  59), intermediate hyperglycaemia (*n*  =  79) or normal glycaemia/no DM (*n*  =  37). Treatment outcome was monitored, and patients were categorized as having a good (cured) or poor (failed, recurrence, died) outcome during treatment and 12 months follow-up. Whole blood transcriptional profiles before, during and at the end of TB treatment were characterized using unbiased RNA-Seq and targeted gene dcRT-MLPA.

**Findings:**

We report differences in whole blood transcriptome profiles, which were observed before initiation of treatment and throughout treatment, between patients with a good versus poor TB treatment outcome. An eight-gene and a 22-gene blood transcriptional signature distinguished patients with a good TB treatment outcome from patients with a poor TB treatment outcome at diagnosis (AUC = 0·815) or two weeks (AUC = 0·834) after initiation of TB treatment, respectively. High accuracy was obtained by cross-validating this signature in an external cohort (AUC = 0·749).

**Interpretation:**

These findings suggest that transcriptional profiles can be used as a prognostic biomarker for treatment failure and success, even in patients with concomitant DM.

**Funding:**

The research leading to these results, as part of the TANDEM Consortium, received funding from the European Community's Seventh Framework Programme (FP7/2007-2013 Grant Agreement No. 305279) and the Netherlands Organization for Scientific Research (NWO-TOP Grant Agreement No. 91214038). The research leading to the results presented in the Indian validation cohort was supported by Research Council of Norway Global Health and Vaccination Research (GLOBVAC) projects: RCN 179342, 192534, and 248042, the University of Bergen (Norway).

**Research in context**

**Evidence before this study**

Tuberculosis (TB), an infectious disease caused by *Mycobacterium tuberculosis*, affects 10 million people annually and is among the deadliest infectious diseases worldwide. Unfortunately, a significant proportion of tuberculosis patients fail to respond to tuberculosis treatment, leading to persistent disease (and spreading of the infection), relapse or even death. Diabetes mellitus as comorbidity in TB patients increases the risk of treatment failure. Identifying individuals with a poor TB treatment outcome early after initiation of treatment is crucial for rapid clinical interventions.

Whole blood transcriptomic biomarkers are promising in identifying individuals with active tuberculosis as well as in the prediction of TB treatment outcome. Previous studies have identified significant differences in the transcriptome of patients with tuberculosis only versus patients with tuberculosis and diabetes comorbidity. However, previously published biomarker signatures of treatment responsiveness have only rarely been tested on tuberculosis patients with concomitant diabetes or hyperglycaemia.

**Added value of this study**

In the present study, we collected whole blood RNA samples from tuberculosis patients with or without hyperglycaemia or diabetes from South Africa and Indonesia. By two independent transcriptomic techniques, RNA-Seq and dcRT-MLPA, we identified transcriptomic profiles discriminating patients who had a good TB treatment outcome from patients with a poor TB treatment outcome. Importantly, we identified eight- and 22-gene signatures to predict TB treatment outcome at diagnosis and at two weeks after initiation of TB treatment, respectively. The signature had a high accuracy in predicting TB treatment outcome in an external Indian validation cohort, including TB patients with or without diabetes. These gene signatures show the potential of transcriptomic biomarkers for early adaptation of treatment in TB patients with different genetic and geographic background, and importantly, in TB patients with concomitant diabetes.

**Implications of all the available evidence**

We showed that TB treatment outcome gene signatures can distinguish patients who will successfully complete TB treatment from patients with treatment failure, even in areas with high diabetes incidence. These signatures may support and accelerate treatment adaptation for TB patients with poor predicted outcomes to treatment. Further longitudinal studies are required to validate the TB treatment outcome signatures in other endemic areas and in TB patients with other comorbidities.

Alt-text: Unlabelled box

## Introduction

With more than 10 million new cases and approximately 1·5 million deaths annually, tuberculosis (TB), which is caused by *Mycobacterium tuberculosis* (*Mtb*), continues to be a major global health threat.^1^ Upon infection with *Mtb*, 5–10% of adults develop active disease during their lifetime and one quarter of the world's population is estimated to be latently infected with *Mtb* (LTBI).^1^ The global TB treatment success rate is only about 85% and even lower in patients with multi-drug resistant TB or with comorbidities like HIV or diabetes mellitus (DM),^1^, ^2^, ^3^ resulting in a significant number of patients with poor clinical outcomes.

DM triples the risk of developing active TB^4^ and increases the risk of poor clearance of the infection following TB treatment.^5^, ^6^, ^7^ In 2020, 0·37 million TB cases were estimated to suffer from DM comorbidity.^1^ Around 85–95% of all DM cases are attributed to type-2 diabetes mellitus (T2DM).^8^ Since global DM prevalence is estimated to rise from 463 million people in 2019 to 700 million in 2045,^9^ in particular in areas where TB is endemic, there is increasing concern about the consequences of the rising DM prevalence for global TB control.^1^ The mechanisms underlying DM-induced TB treatment failure remain, however, poorly understood.

Prediction of TB treatment failure based on sputum-smear microscopy and mycobacterial culture lacks sensitivity^10^ and depends on the quality of sputum samples, which are difficult to collect and are frequently inconsistent in quality.^11^, ^12^, ^13^ As well as more advanced sputum-based diagnostics, monitoring of whole blood transcriptomics may be an additional, complementary but independent, method to monitor treatment responses, possibly with increased sensitivity.^14^ Numerous studies have reported transcriptional biomarker profiles for active TB and response to TB treatment using whole-blood or PBMCs in settings with varying TB incidence.^15^, ^16^, ^17^, ^18^, ^19^, ^20^ In addition, multiple studies have demonstrated the predictive potential of host gene biomarkers in identifying patients at risk of developing active TB, relapse and treatment failure.^21^, ^22^, ^23^, ^24^, ^25^, ^26^, ^27^, ^28^ Together, these studies showed that gene signatures may have utility at predicting TB treatment success versus failure early after TB diagnosis, providing a significant improvement over the currently used, low sensitivity, conversion to negative sputum-based culture testing.^10^ Despite the high incidence of DM and pre-DM among TB patients in TB-endemic settings,^7^^,^^29^, ^30^, ^31^ only a few studies have identified or validated such signatures in TB patients with DM or hyperglycemia.^32^^,^^33^

Characterizing transcriptomic profiles may improve our understanding about immunological pathways that are involved in DM-associated TB pathology, and monitoring treatment success and failure in TB patients with concomitant DM is key to combatting the tuberculosis-diabetes (TB-DM) co-epidemic. Although the blood transcriptome profile of TB-DM patients is more similar to TB patients than to DM patients, suggesting a dominant influence of active TB infection, we and others recently demonstrated significant differences in the blood transcriptome of TB-DM patients compared to TB patients.^32^^,^^33^ Additionally, the transcriptomic profiles of patients with TB-related intermediate hyperglycemia (TBrel-IH) are similar to the profiles of TB-DM patients.^32^ Importantly, we also showed that DM comorbidity lowered the performance of published diagnostic biomarker signatures.^32^ Therefore, there is a need for biomarkers that predict treatment outcome in heterogenous TB populations, including TB-DM patients.

The aim of the current study was to identify a blood transcriptional gene signature to predict TB treatment outcome at an early stage after initiation in a TB population including patients with varying glycaemia and DM status. We combined an unbiased RNA-Seq approach and a selective dcRT-MLPA approach (a multiplex RT-PCR platform) as two independent strategies to identify gene signatures with high discriminatory power to distinguish patients with a good TB treatment outcome from patients with a poor TB treatment outcome. Host gene biomarker profiles to identify TB treatment success or failure could facilitate the evaluation of new TB drugs and improve clinical surveillance of TB patients, even in settings with high DM incidence.

## Methods

### Study participant recruitment, classification and treatment

Adult pulmonary TB patients were recruited between January 2014 and February 2017, as part of the TANDEM project^29^ in two locations: Bandung in Indonesia (UNPAD) and Cape Town in South Africa (SUN) (Supplementary Figure S1). All TB patients were newly diagnosed and microbiologically confirmed, and included people with TB-DM. The TB-DM group included participants with both pre-diagnosed DM and newly identified DM, with new diagnosis based on a laboratory HbA1c test ≥6·5% with a confirmatory HbA1c test ≥6·5% or fasting blood glucose ≥7 mmol/L at TB diagnosis,^29^ followed by a further HbA1c test ≥6·5% after 6 months of TB treatment. The TB patients without DM included people with a normal glycaemic index (laboratory HbA1c <5·7%) at TB diagnosis (“TB-only”). Patients whose HbA1c test results were ≥5.7% and <6.5% were deemed to have intermediate hyperglycaemia. In South Africa, healthy community controls (HC) without TB or DM were also recruited at baseline: HC were all sputum smear and culture negative, had normal chest x-rays and had laboratory HbA1c < 5.7%. The age range and sex balance was similar across the HC and the TB patients, analysed either as all TB patients combined, or for separate treatment outcome groups (Table 1). Multi-drug-resistant TB, HIV positivity, pregnancy, serious co-morbidity and corticosteroid use were exclusion criteria.

**Table 1:** Study participant demographics.

Characteristic	Country	TB Patients			Healthy controls	*P*-value Good vs Poor outcome 2-way comparison	*P*-value Combined TB groups vs HC2-way comparison	*P*-value Good Outcome vs Poor Outcome vs HC3 way comparison
		Good Outcome	Poor Outcome	Combined TB groups				
Total Number of Participants	S Africa	76	18	94	27	-	-	-
	Indonesia	61	20	81	0	-	-	-
	**All**	**137**	**38**	**175**	**27**	-	-	-
Age in years, median (range)	S Africa	46 (22-68)	42 (19-55)	45 (19-68)	42 (30-70)	0.258	0.728	0.485
	Indonesia	49 (25-73)	49 (35-68)	49 (25-73)	-	0.96	-	-
	**All**	**47 (22-73)**	**47 (19-68)**	**47 (19-73)**	**42 (30-70)**	**0.619**	**0.1286**	**0.2801**
Sex, % male (No. male/ female)	S Africa	58 (44/32)	67 (12/6)	60 (56/38)	52 (14/13)	0.495	0.474	0.615
	Indonesia	53 (32/29)	65(13/7)	56 (45/36)	-	0.258	-	-
	**All**	**56 (76/61)**	**66 (25/13)**	**58(101/74)**	**52 (14/13)**	**0.212**	**0.567**	**0.385**
Number with Diabetes / Intermediate Hyperglycaemia / Normal glycaemia (%)	S Africa	13/49/14	4/11/3	17/60/17	0/0/27	0.8785	<0.0001	<0.0001
	Indonesia	31/16/14	11/3/6	42/19/20	-	0.559	-	-
	**All**	**44/65/28**	**15/14/9**	**59/79/37**	**0/0/27**	**0.541**	**<0.0001**	**<0.0001**
HbA1c median (range)	S Africa	6.0 (4.9-14.3)	6.0 (4.8-14.1)	6.0 (4.8 – 14.3)	5.3 (4.8-6.4)	0.614	<0.0001	<0.0001
	Indonesia	8.15 (4.9-17.1)	7.1 (5.1-14.1)	7.8 (4.9 – 17.1)	-	0.561	-	-
	**All**	**6.0 (4.9-17.1)**	**6.1 (4.8-14.1)**	**6.0 (4.8 – 17.1)**	**5.3 (4.8-6.4)**	**0.989**	<0.0001	<0.0001
BMI at TB diagnosis:, median (range)	S Africa	18.7 (13.9-32.3)	18.3 (13.7-31.2)	18.7 (13.9-32.3)	23.2 (17.4-45.2)	0.903	<0.0001	0.0001
	Indonesia	19.7 (13.8-33.3)	18.8 (16.3-27.3)	19.6 (13.8-33.3)	-	0.843	-	-
	**All**	**19.1 (13.8-33.3)**	**18.8 (13.7-31.2)**	**19.0 (13.7-33.3)**	**23.7 (17.4-45.2)**	**0.835**	<0.0001	<0.0001
TTP (days) at TB diagnosis: Median (range) (missing values)	S Africa	6 (1-21) (18)	6 (3-21) (3)	6 (1-21) (21)	N/A	0.820	-	-
Smear Grade at diagnosis number: 3+/2+/1+/scanty/negative	Indonesia	16/23/14/2/6	8/4/5/2/1	24/27/19/4/7	N/A	0.364	-	-
Sputum conversion at Month 2: number yes/no (missing values)	S Africa	49/19 (8)	10/6 (2)	59/25 (10)	N/A	0.452	-	-
	Indonesia	44/15 (2)	10/8 (2)	54/23 (4)	N/A	0.123	-	-
	**All**	**93/34 (10)**	**20/14 (4)**	**113/48 (14)**	**N/A**	0.103	-	-
Outcome classification: Cured /Recurrence/Failed/Died	S Africa	76/0/0/0	0/4/10/4	76/4/10/4	N/A	<0.0001	-	-
	Indonesia	61/0/0/0	0/2/16/2	61/2/16/2	N/A	<0.0001	-	-
	**All**	**137/0/0/0**	**0/6/26/6**	**137/6/26/6**	**N/A**	**<0.0001**	-	-
RNASeq subset	S Africa	26	6	32	0	-	-	-
	Indonesia	23	8	31	0	-	-	-
	**All**	**49**	**14**	**63**	**0**	-	-	-
MLPA subset	S Africa	76	18	94	27	-	-	-
	Indonesia	58	19	77	0	-	-	-
	**All**	**135**	**37**	**172**	**27**	-	-	-

Combined TB Groups is the combination of TB patients with a Good or Poor Outcome. Continuous variables were compared by Mann-Whitney U test (2 groups) or Kruskal-Wallis test (3 groups); non-continuous variables by Chi-square test.

TB patients received standard first line TB treatment according to WHO Guidelines. Microbiological measures recorded at baseline and throughout treatment included sputum smear and culture, with time to positivity (TTP) in mycobacteria growth indicator tubes (MGIT) also assayed in South Africa. TB patients were classified based on their TB treatment outcome: “poor TB treatment outcome” included those patients who died, failed initial treatment (remained sputum positive at six months) or experienced TB-recurrence in the 12 month clinical follow-up period post treatment, whilst those with “good TB treatment outcome” had successful TB treatment without subsequent recurrence. Patients for whom the outcome data were missing were not included in downstream analyses. Most TB-DM patients received local standard of care DM treatment following national guidelines outside of the TANDEM study, which largely involved metformin and glibenclamide prescription, whilst a subgroup in Indonesia received more intensive education and counselling, glucose and HbA1c monitoring and treatment adjustment through TB treatment as part of a pragmatic randomised control trial,^34^ in which they were assessed at weeks 1, 2 and 4 and then monthly throughout TB treatment, and treatment optimised at each visit, leading to better HbA1c control at 6 months in this group.

### External validation data

Data from a prospective cohort study of adult pulmonary TB cases were used for external validation.^28^ The cohort consists of pulmonary TB patients that were recruited in Palamaner and Kuppam Taluks, Chittoor district, Andhra Pradesh, India between September 2010 and April 2012. Pulmonary TB was radiologically confirmed. Patients received standard TB treatment and were followed for six months. For this study, data from 67 participants were available. This cohort was constituted of 55 (82%) males and 12 (18%) females with a mean age of 43 (18–75) years. Among the 67 patients, 45 (67%) patients had successful TB treatment (“good TB treatment outcome”), while 22 (33%) failed treatment (“poor TB treatment outcome”). Diabetes was recorded in 9 (13%) participants, all of whom had successful TB treatment.

### Ethics statement

The study was approved by the London School of Hygiene & Tropical Medicine Observational Research Ethics Committee (6449), the SUN Health Research Ethics Committee (N13/05/064) and the UNPAD Health Research Ethics Committee, Faculty of Medicine, Universitas Padjadjaran (number 377/UN6.C2.1.2/ KEPK/ PN), and participants gave written informed consent.

### RNA sample collection and extraction

Patient samples were collected prior to initiation of treatment (diagnosis), at weeks 2, 4, 8, 16 and 24 through treatment, and at 12 months after TB diagnosis (6 months after treatment completion), and from HC at baseline only. Venous blood (2·5ml) was collected into PAXgene Blood RNA Tubes (PreAnalytiX). Total RNA was extracted using RNeasy spin columns (Qiagen) and quantified by Nanodrop (Agilent). The LabChip GX HiSens RNA system (PerkinElmer) was used for quality assessment of samples processed by RNA-Seq.

### Unbiased RNA-Seq of global gene expression

Samples collected at TB diagnosis and weeks 2, months 2, and months 6 from the first 63 participants recruited were analysed by RNA-Seq (Table 1). Libraries were generated using the poly-A tail Bioscientific NEXTflex-Rapid-Directional mRNA-Seq method with the Caliper SciClone. Single-end sequencing was performed using the NextSeq500 High Output kit V2 (Illumina) for 75 cycles. Sequence data from FASTQ files were aligned to the Human g1kv37 reference genome, using STAR (v2.5.1b).^35^ Quality control was performed with FastQC,^36^ while transcript quantification was performed using HT-seq count (v0.61)^37^: lowly expressed transcripts (<50 counts across all samples), were removed from the downstream analysis. RNA-Seq data were normalised using DESeq2 (v1.30.0).^38^

### Dual-color reverse-transcriptase multiplex ligation-dependent probe amplification (dcRT-MLPA)

Dual-color reverse-Transcriptase Multiplex Ligation-dependent Probe Amplification (dcRT-MLPA) was performed on all samples to identify blood transcriptional profiles as described previously.^39^ Brief descriptions are provided in the Supplementary Information. RT primers and half-probes were designed by Leiden University Medical Centre (LUMC, Leiden, The Netherlands) and encompassed sequences for 144 selected key immune-related genes to profile the innate, adaptive and inflammatory immune responses (Supplementary Table S1), and four housekeeping genes (*GAPDH, ABR, GUSB, B2M*). Genes with an adjusted *P*-value <0·05 (Benjamini-Hochberg^40^) and a log2-fold change (FC) <-0·6 and >0·6 were considered differentially expressed genes (DEGs). Genes that were below the detection limit in >90% of the samples per cohort were excluded from analysis.

### Data analysis and statistics

Statistical analyses to compare participant demographics were carried out using GraphPad Prism 8 software (Graphpad Software, San Diego, CA, USA). Data for most variables were not normally distributed, as determined by the D'Agostino & Pearson and the Shapiro-Wilk tests, thus non-parametric statistical comparison methods were employed for analysis of the study participants, For continuous measures, a Mann-Whitney U-test was used when comparing two groups and a Kruskal-Wallis test when comparing three groups. For non-continuous measures, the Chi-square test was used. *P*-values <0·05 were considered significant.

Molecular Degree of Perturbation (MDP) analysis was performed to quantify the molecular distance of samples within a group compared to a reference group (“healthy controls” or “diagnosis”). MDP scores were calculated by R using the *mdp* R package,^41^ and differences between the mean ranks of the groups were assessed by Mann-Whitney U test followed by Benjamini-Hochberg False discovery correction.^40^ Cell population estimates were calculated using the Cell-type Computational Differential Estimation cellCODE^42^ R package which enables the prediction of cellular composition without external measurement, and which would allow prediction of cell-specific gene expression if samples were heterogeneous: the Immune Response In Silico:IRIS^43^ and Differentiation Map:DMAP^44^ data sets were used as references in CellCODE analysis. Modular analysis was performed using the R package tmod^45^ and its HGtest method, with DEGs used as the foreground and all genes used as the background signal. Modular analysis aims to reduce the complexity of transcriptomic datasets, grouping together sets of genes which are co-expressed and behave in a similar manner across experimental designs.

Principal Component Analysis (PCA) and Pearson correlation (R package stats, function prcomp and cor.test, respectively) were used to evaluate the influence of sex, age, BMI, and HbA1c levels on the gene expression.

Differential expression analysis (DEA) was performed in the RNA-Seq dataset in R using the MaSigPro package^46^ to characterise longitudinal differential gene expression of genes measured by RNA-Seq: this followed a two-step regression method, firstly using the least squared technique and also performing a false discovery rate correction, and secondly a stepwise regression to find genes with significant temporal expression changes, significant differences between clinical groups and to find clusters of genes with similar expression behaviour. A quadratic regression model was executed due to the number of timepoints analysed.

Longitudinal DEA of genes measured by dcRT-MLPA was assessed by means of linear mixed models for repeated measures over time using lme4 package in R.^47^ A Benjamini-Hochberg False discovery correction was performed, with an adjusted *P*-value of <0·05 deemed significant. Non-parametric Mann-Whitney U-test followed by Benjamini-Hochberg correction was performed to identify DEGs between patients who had a good and poor TB treatment outcome. Correlations were evaluated using Pearson's correlation coefficient.

TB treatment outcome signatures based on dcRT-MLPA data were identified in TB patients from South Africa and Indonesia using Recursive Feature Elimination (RFE)^48^ and Random Forest (RF). Because the number of patients with a good TB treatment outcome was considerably larger than those with a poor TB treatment outcome (poor, *n*  =  38; good, *n*  =  134), a random down-sampling technique as well as a Synthetic Minority Oversampling Technique (SMOTE) were applied to balance the classes (i.e. “good TB treatment outcome” and “poor TB treatment outcome”) of the dataset.^49^ RF was performed as machine learning algorithm on the dataset including the selected genes and the performance of gene signatures was evaluated by Leave-One-Out Cross Validation (LOOCV).^50^^,^^51^ We assessed the classifying performance of the model by evaluating Receiver Operating Characteristic (ROC) curve and Area Under the ROC Curve (AUC) with 95% Confidence Interval (CI). DeLong test was used to compare correlated ROC curves. An extended description of the data-analysis methods is provided in the Supplementary Information.

### Role of funders

Funders had no role in study design, data collection, data analyses, data interpretation, writing of the report and decision to submit the paper for publication.

## Results

### Study design and cohort

Pulmonary TB patients were recruited into the prospective longitudinal study in South Africa (*N*  =  94) and Indonesia (*n*  =  81), and followed up through standard treatment and for the following 12 months. Altogether, 38 TB patients of the 175 recruited had a “poor TB treatment outcome”, with 6 patients dying, 26 failing treatment (based on continued sputum smear or culture positivity at month 6), and 6 experiencing recurrences in the subsequent 12 months (Supplementary Figure S1). The “poor TB treatment outcome” rates were similar in the two sites (Table 1). The median age of the patients was equal in patients with either a good or poor TB treatment outcome (median  =  47 years), with a higher proportion of males with a poor TB treatment outcome (67%) than a good TB treatment outcome (56%). While there was a slightly higher proportion of patients with DM in the poor TB treatment outcome group (15/39; 38%) than the good TB treatment outcome group (44/137; 32%), the difference in DM status across the outcome groups was not statistically significant in Chi squared test (*P*  =  0.55; Table 1). By definition, there were significant differences in DM status and HbA1c between TB patients and HC, as the latter were recruited based on their normal glycaemic status. There was no evidence that those who had a poor TB treatment outcome had more severe TB at diagnosis, with similar sputum bacterial loads (as measured by TTP) in TB patients from South Africa and similar sputum smear grade in Indonesia across the good and poor TB treatment outcome groups. The BMIs in the TB patients with good or poor treatment outcome were not significantly different to each other (*P*  =  0.835), but were highly significantly lower (*P* < 0.0001) than the HC group when tested as separate groups or as a combined TB patient group, which is expected as TB patients are often underweight.

### The transcriptomic response in patients with a poor and good TB treatment outcome

**Figure 1:**
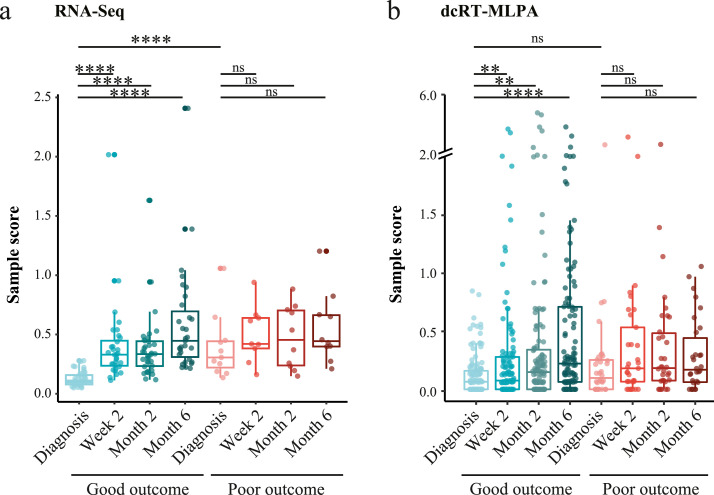
**MDP plots representing the change in gene expression perturbation in TB patients categorized based on TB treatment outcome**. Full blood transcriptomes from TB patients who had a good or poor TB treatment outcome were determined by (a) RNA-Seq and by (b) dcRT-MLPA. The extent of overall difference in gene expression, relative to the median of expression at diagnosis in those who had a good TB treatment outcome, was calculated for individual patients at the timepoints shown. The bars and whiskers show the median and data within the Q_1_-1·5 x inter quartile range (IQR) and Q_3_+1·5 x IQR interval. Differences were significant by Mann-Whitney U-test with Benjamini-Hochberg correction for multiple testing. * *P* < 0·05, ** *P* < 0·01, *** *P* < 0·001, **** *P* < 0·0001.

The holistic unbiased analysis of gene expression in TB patients with good or poor TB treatment outcomes by RNA-Seq approach was performed on a subset of study participants, who were the first 63 recruited participants (Table 1). Molecular Degree of Perturbation (MDP) analysis revealed that there were significant changes in global gene expression in patients with a good TB treatment outcome continuously through TB treatment, reflecting treatment response (Figure 1a). Gene expression perturbation was also evident in patients who had a poor TB treatment outcome, although the sample score was higher at diagnosis compared to patients who had a good TB treatment outcome. This represents differences at the transcriptomic level between patients with a good versus a poor TB treatment outcome, already evident before initiation of TB treatment. This was followed by less change over time in response to TB treatment in the poor TB outcome group.

Next, we focused our molecular distance analysis on 144 TB-associated genes as measured by dcRT-MLPA, which was performed on all study participants (*n*  =  199) (Table 1). There were significant changes in global gene expression already observable two weeks after initiation of TB treatment in patients with a good TB treatment outcome, but not in patients with a poor TB treatment outcome (Figure 1b), reflecting a delayed TB treatment response in the latter group. Gene expression perturbation normalized towards levels of healthy controls throughout treatment in patients with a good TB treatment outcome, but not in patients with a poor TB treatment outcome (Supplementary Figure S2). However, despite the substantial treatment response in patients with a good TB outcome, gene expression perturbation did not completely normalize to levels of healthy controls.

Together, these data suggest that there was a different biosignature in those with good versus poor TB treatment outcomes, which was reflected by transcriptomic differences before initiation of TB treatment and by a delayed response to TB treatment in patients with a poor TB treatment outcome compared to patients with a good TB treatment outcome.

### Global differential expression analysis in patients with good or poor TB treatment outcome

**Figure 2:**
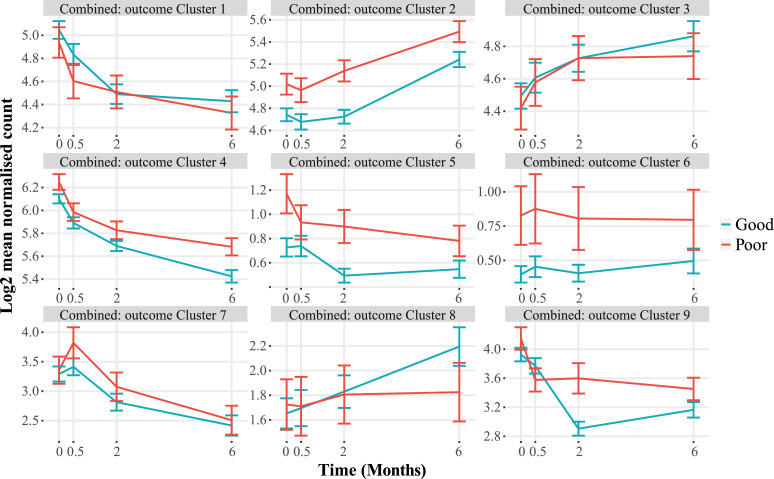
**MaSigPro analysis of TB patients with good or poor TB treatment outcome, across combined South African and Indonesian cohorts**. Plots show hierarchical clusters of genes in patients with a good (blue) or poor (red) TB treatment outcome. Bars show mean ± 1 SEM. Data were filtered to remove lowly abundant transcripts prior to analysis.

The changes in gene expression in the RNA-Seq dataset through time and between the patients with a good or poor TB treatment outcome were analyzed by MaSigPro, an R package designed for longitudinal RNA-Seq data, initially in the pooled South African and Indonesian cohorts. The MaSigPro regression modelling tool treats time as a quantitative variable, so as well as differentially expressed genes (DEGs) being detected, the changes in trends and magnitude are also included. Thus using MaSigPro, we could determine the change in gene expression over time and also between different treatment groups (Figure 2, Supplementary Table S4). The genes differentially expressed through treatment in the pooled analysis separated into nine clusters, with variable patterns of expression over time and between TB patients with good or poor TB treatment outcome. Some clusters^2^^,^^5^^,^^6^ contained genes which were different between the groups at all time points, whereas other clusters^1^^,^^3^, ^4^, ^5^^,^^7^, ^8^, ^9^ were similar at some timepoints and more divergent at others (Figure 2, Table 2). Similarly, cluster analysis of gene expression in the cohorts *separately* revealed nine gene clusters, with gene clusters increasing (South Africa: 1,2,3,5, Indonesia: 2,5,7) or decreasing (South Africa: 4,6,7,8, Indonesia: 1,3,4,6,8,9) through time (Supplementary Figure S3, Supplementary Table S2 and S3), and with higher expression in TB patients with a poor outcome in gene clusters (South Africa: 1,3,4,5,7,8,9, Indonesia: 2,6,8,9) and with a good outcome in gene clusters (South Africa: 2,6, Indonesia: 1,3,4). Importantly, these differences in gene expression through time were observed in all TB patient groups, irrespective of their DM status.

**Table 2:** Clusters of genes differentially expressed between TB patients with good or poor TB treatment outcomes to TB treatment in MaSigPro analysis of combined RNA-Seq data from South Africa and Indonesia.

Cluster number	Overall pattern	Number of gene transcripts	Gene function				Top Functions from g:Profiler^b^with adjusted *P* < 0·05
			Protein coding	Processed transcript	Pseudo-gene	Regulatory RNAs^a^	
1	↓ through treatment; Higher in Good at M0.5	26	20	1	0	5	GO:MF – Opsonin Binding; GO:CC – Intracellular Vesicles; Endomembrane System
2	↑ through treatment; Higher in Poor throughout	14	13	0	0	1	GO:BP – B cell receptor (BCR) signaling; GO:CC – BCR complex KEGG – BCR signaling; primary immunodeficiency; REAC – BCR signaling WP – BCR signaling CORUM – CIN85-BLNK complex
3	↑ through treatment; Higher in Good at M6	25	17	2	0	6	*No significant results*
4	↓ through treatment; Higher in Poor throughout	47	37	2	2	6	GO:CC – Arp2/3 complex; KEGG – Shigellosis; E.coli, Yersinia, Salmonella infection; Endocytosis; REAC – Ephrin signaling; Rho GTPAses activate WASPs and WAVEs; TF – ZNF544 CORUM – Arp2/3 complex
5	Small ↓ through treatment; Higher in Poor throughout	11	6	1	1	3	GO:MF – L-tyrosine transmembrane transporter activity; GO:BP – positive regulation of fatty acid transport.
6	No change; Higher in Poor throughout	4	0	0	3	1	*No significant results*
7	↑ to M0.5, then ↓; Higher in Poor at M0.5	4	4	0	0	0	GO:BP – mitotic cell cycle process WP – Retinoblastoma Gene in Cancer
8	↑ in Good through treatment; No change in Poor	7	3	0	2	2	GO:MF - RNA polymerase III activity
9	↓ through treatment; Much greater change Good	10	10	0	0	0	GO:MF – immunoglobulin receptor binding GO:BP – phagocytosis, recognition; complement activation, classical pathway; immunoglobulin mediated immune response; B cell activation GO:CC – immunoglobulin complex; E/C space; plasma membrane REAC – Classical antibody-mediated complement activation; FCGR activation; phagocytosis.

a Retained introns, Antisense, LncRNA, miRNA, nonsense-mediated decay, sense overlapping, sense intronic, snoRNA,

b Redundant G:Profiler results are not shown.

The number of transcripts within each cluster in the combined pooled cohort analysis ranged from 4 to 47 (Table 2), with the majority of genes identified in all clusters encoding proteins. There were also various regulatory transcripts in some clusters, including long non-coding RNAs, miRNA, snoRNA, retained introns, as well as antisense, nonsense-mediated decay, overlapping senses and sense intronic transcripts. To understand the biological function of the differentially expressed genes (DEGs), the transcripts within each cluster were analysed using the g:COST tool within the g:Profiler application,^52^ to determine significant enrichment of genes in Gene Ontology (GO) molecular function, cellular component and biological process categories, as well as in curated biological pathways from KEGG and Reactome databases and the CORUM protein database. Genes in cluster 2 were largely involved in B cell receptor signalling, seen in the GO and pathway analyses, and these were more highly expressed in people who had a poor TB treatment outcome, with increasing expression through treatment. This upregulation of genes involved in B cell function, particularly those involved in earlier stages of B cell development, was not related to the overall number of B cells in the samples, as predicted from the samples using CellCODE analysis package which showed that there were no significant differences in the proportions of any of the predicted cell types (corrected P>0.05; Supplementary Figure S4). Cluster 9 was predominantly composed of immunoglobulin transcripts, whose expression decreased much more substantially in patients with a good TB treatment outcome. The largest gene cluster^4^ was enriched with genes involved in actin remodelling, including the Arp 2/3 complex, and in pathways related to infections with bacteria such as Shigella, *E. coli*, Yersinia and Salmonella. Cluster 7 contained genes related to mitotic cell division, and these were more highly expressed in patients with a poor TB treatment outcome (Table 2). These analyses were also performed using the DAVID online tool,^53^ and similar results were obtained (not shown). The DEGs found in the combined and separate cohort MaSigPro analyses were used as a foreground against all genes in a modular analysis using the Tmod package, which gives biological function to a gene list. It showed an upregulation of genes involved in B cell function in good versus poor TB treatment outcomes, in both the Indonesian and South African cohorts (Supplementary Table S5).

### Differential expression analysis of focused gene expression in patients with good or poor TB treatment outcome

**Figure 3:**
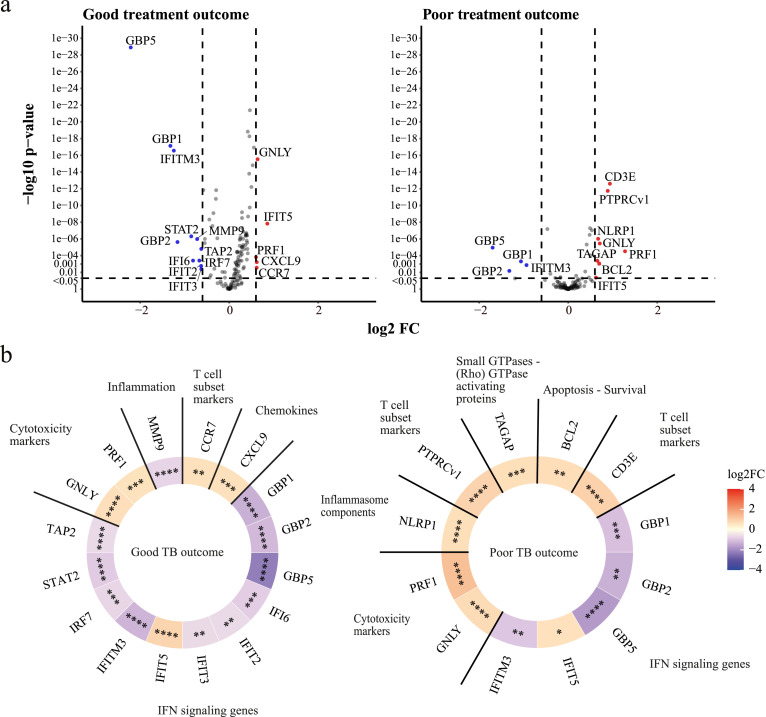
**DEA of all TB patients from the pooled (South African and Indonesian) cohorts categorized based on TB treatment outcome compared to their gene expression levels at diagnosis**. (a) Volcano plots representing DEGs regulated during TB treatment of TB patients who had a good TB treatment outcome (left panel) or a poor TB treatment outcome (right panel). The y-axis scales of the plots are harmonized per TB treatment outcome. -log_10_-transformed *P*-values are plotted against log_2_ FC. Genes with *P* < 0·05 and log_2_ FC<-0·6 or >0·6 were labelled as DEGs. (b) Heatmaps displaying log_2_ FC of the DEGs and corresponding gene modules. The saturation of color represents the magnitude of differential expression. Differences were significant by means of linear mixed models. * *P* < 0·05, ** *P* < 0·01, *** *P* < 0·001, **** *P* < 0·0001.

Next, we focused our DEA on 144 genes that previously have been associated with TB^39^ using dcRT-MLPA (Supplementary Table S6). We decided to analyze longitudinal expression of genes (Figure 3) because no significant DEGs were detected by directly comparing patients with a good versus a poor TB treatment outcome at the indicated timepoints (Supplementary Figure S5). Kinetic profiling of DEGs identified 16 DEGs in patients with a good TB treatment outcome and 12 DEGs in patients with a poor TB treatment outcome. Genes associated with active TB^15^^,^^20^^,^^54^ or risk of developing TB^22^ were substantially downregulated (*GBP1, GBP2, GBP5,* and *IFITM3*) or upregulated (*GNLY* and *PRF1*) over time in TB patients regardless of their TB treatment outcome, reflecting transcriptomic response to TB treatment (Figure 3A and Supplementary Figure S6). Other genes associated with active TB were significantly down- or upregulated (*STAT2, MMP9, IRF7, IFI6, IFIT2, IFIT3,* and *CCR7*) during TB treatment in patients who had a good TB treatment outcome, but not in patients who had a poor TB treatment outcome, while genes such as *CD3E, PTPRCv1, NLRP1, BCL2* were upregulated in patients with poor TB outcome but not in patients with good TB outcome, confirming that altered changes in patients with a poor outcome could be observed using this methodology, despite the smaller sample size.^15^^,^^39^^,^^54^^,^^55^ The expression of *TAGAP,* previously associated with active TB,^55^ was significantly increased during TB treatment in patients who had a poor TB treatment outcome. A high correlation between DEGs of patients who had a poor TB treatment outcome and DEGs of patients who had a good TB treatment outcome could be detected (R = 0·87, *P* < 0·0001), highlighting the challenge of discriminating patients with a good versus a poor TB treatment outcome based on single genes (Supplementary Figure S7). Modular analysis showed that the gene profile of regulated genes was dominated by genes in the interferon (IFN) signaling pathway, especially in patients who had a good TB treatment outcome (Figure 3b). The longitudinal expression of DEGs identified by dcRT-MLPA showed a significant correlation with genes measured by RNA-Seq, highlighting the validity and reproducibility of our approach (Supplementary Figure S8).

### Identification of a signature predicting TB treatment outcome

Machine learning algorithms were implemented on data obtained at each time point to develop biomarker panels to predict TB treatment outcomes at different stages of TB treatment. First, we aimed to identify gene signatures from RNA-Seq analysis on a subset of subjects, but we found a low performance of gene signatures generated on diagnosis, week two and month six (AUC = 0·625, AUC = 0·667 and AUC = 0·615, respectively) to predict TB treatment outcome, potentially due to a low number of patients in the training and test set (Supplementary Figure S9A). The best performing model was built on month two resulting in an AUC of 0·8667 (Supplementary Figure S9A, Supplementary Table S7). We also tested an active TB disease biomarker signature, namely the three-gene Sweeney signature,^20^ to determine whether it resolved significantly more in those with a good TB treatment outcome than in those with a poor TB treatment outcome. This signature has previously been shown to persist in patients with persistent lung inflammation.^26^ However, in our RNA-Seq data, this signature revealed an AUC of 0·5333 (Supplementary Figure S9B) highlighting that the process behind poor TB treatment outcome cannot be predicted by expression of these three genes.

**Figure 4:**
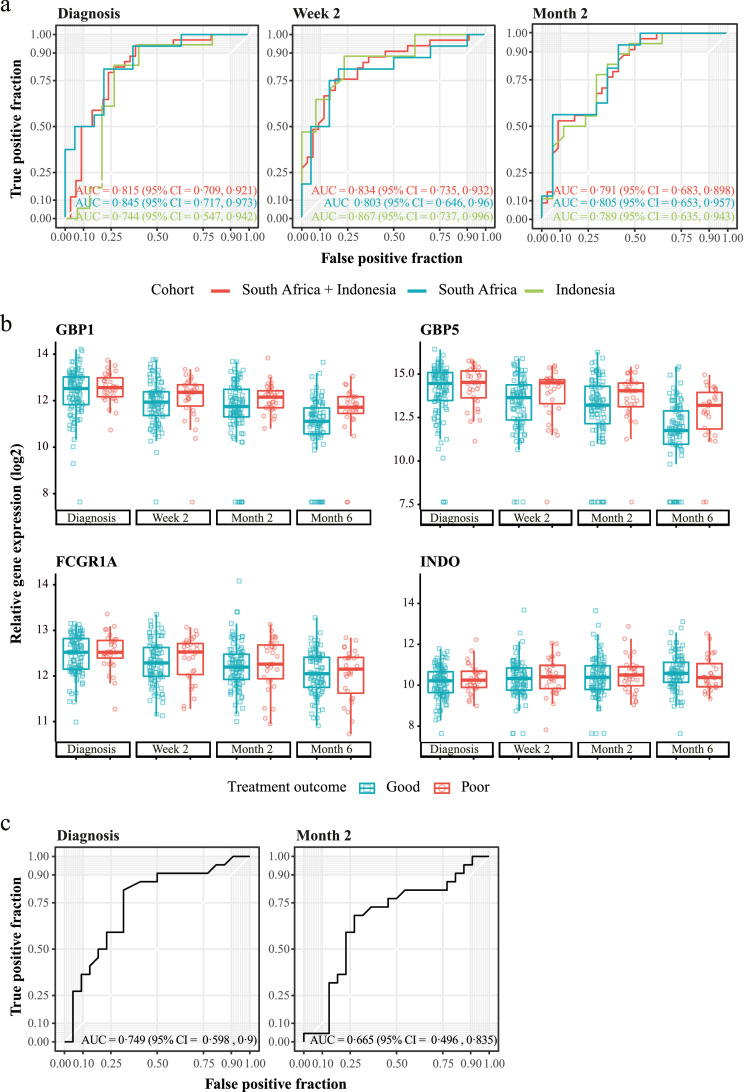
**Prediction of TB treatment outcome in dcRT-MLPA data from peripheral blood.** (**a**) ROC curves showing the predictive power of the gene signatures identified in the balanced pooled cohort (South Africa and Indonesia) to classify TB patients at diagnosis (left panel), two weeks (middle panel) or two months (right panel) after initiation of TB treatment into patients who had a good TB treatment outcome and patients who had a poor TB treatment outcome, using the RFE-RF model and LOOCV. The dataset was balanced by down-sampling to encompass the same number of individuals with poor and good TB treatment outcome (diagnosis, *n* = 34; week two, *n* = 33; month two, *n* = 34). (b) Gene expression kinetics of the single genes encompassing the diagnosis or week two gene signatures predicting TB treatment outcome in the pooled cohort. Box plots depict *GAPDH*-normalized, log_2_-transformed median gene expression values and the IQR, while the whiskers represent the data within the Q_1_-1·5xIQR and Q_3_+1·5xIQR interval. (**c**). ROC curves showing the predictive power of the gene signatures identified in the balanced pooled cohort (South Africa and Indonesia) to classify TB patients from an external validation cohort (India) at diagnosis (left panel) or two months (right panel) after initiation of TB treatment into patients who had a good TB treatment outcome and patients who had a poor TB treatment outcome. The dataset was balanced by down-sampling to encompass the same number of individuals with poor and good TB treatment outcome (diagnosis, *n* = 22; month two, *n* = 22). Abbreviations: AUC, area under the curve; CI, confidence interval.

Next, we aimed to identify early correlates of TB treatment outcome by implementing machine learning algorithms on gene expression as measured by dcRT-MLPA. We focused our analysis on the identification of gene predictors at diagnosis and at week two that could possibly be used in future studies to predict the occurrence of poor or good TB treatment outcome before or early after TB treatment initiation, first by down-sampling the good TB treatment outcome class. The top eight ranked genes (*GBP1, FCGR1A, STAT1, IFITM3, BCL2, CCL4, TLR9, CD274*) from the diagnosis signature were used for RF machine learning model implementation (Table 3). Excitingly, the signature had a high predictive power to classify good and poor TB treatment outcome both in all TB patients irrespective of their DM condition and also only in the TB with concomitant diabetes group separately (TB-DM) (AUC = 0·815 and AUC = 0.792, respectively), and this was evident already before TB treatment initiation (Figure 4A and Supplementary Figure 10). Furthermore, the gene signature showed high performance in all TB patients from the separate cohorts (South Africa, AUC = 0·845; Indonesia, AUC = 0·744). By using this signature, we performed PCA and Pearson correlation analysis to verify that parameters such as Sex, Age, HbA1c levels, and BMI did not have influence on the gene expression perturbation (Supplementary Figures 11–14). Next, we investigated whether accuracy could be improved by predicting TB treatment outcome after initiation of TB treatment, thus measuring the early treatment response. We identified a 22-gene signature to predict TB treatment outcome at two weeks after initiation of TB treatment (Table 3). The performance of the week two signature in predicting TB treatment outcome was slightly improved (pooled cohorts, AUC = 0·834; Indonesia, AUC = 0·803; South Africa, AUC = 0·867; TB-DM, AUC = 0.92) compared to the diagnosis signature, especially in patients from the Indonesian cohort (AUC = 0·867 versus AUC = 0·744 at diagnosis). Furthermore, we identified a 14-gene month two signature, however this demonstrated a slightly lower accuracy in predicting TB treatment outcome compared to the diagnosis and week two gene signatures (pooled cohorts, AUC = 0·791; Indonesia, AUC = 0·789; South Africa, AUC = 0·805), but not in TB-DM (AUC = 0.875). No significant differences were detected between models, comparing week two versus diagnosis, month two versus diagnosis, and month two versus week two using the DeLong test for correlated ROC curves (all TB groups with no discrimination between DM conditions: *P*-value = 0·7986, 0·7271, 0·5626, respectively; TB-DM: *P*-value = 0.3434, 0.5353, 0.6553).

**Table 3:** dcRT-MLPA Gene signatures Good versus Poor obtained by pooling the study groups and the cohorts.

Diagnosis signature	
Gene	Module
**GBP1**	IFN signaling genes
**FCGR1A**	IFN signaling genes
STAT1	IFN signaling genes
IFITM3	IFN signaling genes
BCL2	Apoptosis - Survival
CCL4	Treg associated genes
TLR9	Pattern recognition receptors
CD274	IFN signaling genes

Week two signature	
Gene	Module
**GBP5**	IFN signaling genes
**INDO**	IFN signaling genes
**GBP1**	IFN signaling genes
**BMP6**	Cell growth - proliferation
CXCL9	Chemokines
GATA3	Th2 associated genes
**FCGR1A**	IFN signaling genes
**MMP9**	Inflammation
PTPRCv1	T cell subset markers
SPP1	Inflammation
CD3E	T cell subset markers
ASAP1	Small GTPases - (Rho) GTPase activating proteins
IL5	Th2 associated genes
TNFRSF1B	Apoptosis - Survival
NLRP2	Inflammasome components
MRC1	Pattern recognition receptors
NLRP6	Inflammasome components
IL22RA1	Th17 associated genes
VEGF	Cell growth - proliferation
KIF1B	Intracellular transport
CCL19	Chemokines
CD209	Pattern recognition receptors

Month 2 Signature	
Gene	Module
BLR1	G protein-coupled receptors
**BMP6**	Cell growth - proliferation
CCL13	Chemokines
**GBP1**	IFN signaling genes
GBP2	IFN signaling genes
**GBP5**	IFN signaling genes
IFI16	IFN signaling genes
IL9	Th9 associated genes
**INDO**	IFN signaling genes
**MMP9**	Inflammation
NOD2	Pattern recognition receptors
OAS3	IFN signaling genes
PTPRCv2	T cell subset markers
TAP1	IFN signaling genes

Genes that appeared in more than one gene signature (diagnosis, week two or month two) are shown in bold. Gene signatures were obtained by down-sampling the majority class (good TB treatment outcome).

Since we detected differences in the kinetics of gene expression of patients who had a good TB treatment outcome versus patients with a poor TB treatment outcome (Figure 1), we next assessed whether a “delta” gene signature, by subtracting week two values from diagnosis, could improve the predictive performance. The delta signature encompassed seven genes (*GNLY, MRC1, GBP5, NLRP1, FLCN1, ZNF532,* and *IFIT2*) and slightly improved predictive performance (pooled cohorts, AUC = 0·849; South Africa, AUC = 0·839 and Indonesia, AUC = 0·872) compared to the week two and diagnosis signatures (Supplementary Figure S15, Supplementary Table S8). Multiple genes were included in more than one gene signature (Supplementary Figure S16a), of which four genes (*GBP1, GBP5, FCGR1A, INDO*) are shown in Figure 4b. Next, we validated performance of the diagnosis signature and month two signature on an independent Indian validation cohort,^28^ which like our cohorts, included diabetic patients. Our diagnosis gene signature had high predictive power on the Indian validation cohort (AUC = 0·749) (Figure 4c). The week two and delta signatures could not be validated on the Indian cohort, because samples were not collected two weeks after initiation of TB treatment in this cohort. Importantly, three genes (*CD3E, PTPRCv1, NOD2*) that were included in our gene signatures, were also part of gene signatures described by Sivakumaran et al. (Supplementary Figure S16b). Finally, we assessed whether gene signatures with improved performance could be obtained by applying SMOTE^49^ as an alternative sampling technique. A diagnosis SMOTE gene signature was obtained that showed overlap with the diagnosis gene signatures obtained by random down-sampling (Supplementary Table S9, Supplementary Figure S16C). The SMOTE signature produced a high degree of accuracy in discriminating patients with a good TB treatment outcome from patients with a poor TB treatment outcome, but performed with lower accuracy compared to the diagnosis signature obtained by random down-sampling (pooled cohorts, AUC = 0·728; South Africa, AUC = 0·695; Indonesia, AUC = 0·765) (Supplementary Figure S17). The diagnosis SMOTE signature exerted a similar predictive capacity on the external Indian cohort compared to the down-sampling signature (SMOTE, AUC = 0·704; down-sampling, AUC = 0·749).

Taken together, we identified gene signatures with high predictive power on TB treatment outcome, irrespective of DM as comorbidity, in patients from South Africa and Indonesia and in patients from the external Indian validation cohort.

## Discussion

In this study, we identified peripheral blood transcriptional signatures which predict TB treatment success and failure in a TB cohort with patients with varying hyperglycaemia or DM. Previous studies developing biomarker signatures of TB treatment success, recurrence or failure^24^, ^25^, ^26^^,^^56^ did not include people with DM comorbidity, and we have previously found that concomitant DM impairs existing TB diagnosis signature accuracy.^32^ Here we showed DM also affects existing TB treatment-response biomarker signatures in the RNA-Seq dataset, suggesting that they should be derived with cohorts including this population, and our data could be used to test validity of other putative biomarker signatures, such as the RESPONSE5 signature,^25^ in this population.

Our whole cohort dataset, from which we generated TB treatment outcome signatures, was derived using our dcRT-MLPA gene set, which did not contain most of the genes reported in previous signatures, except *GBP5*, which was included in our week two and month two gene signatures. Sivakumaran et al.^28^ recently reported baseline and month two gene signatures predicting TB treatment outcome at six months after initiation of TB treatment, using the same material (whole blood), technique (dcRT-MLPA) and gene set. Notably, our TB treatment outcome gene signatures showed some overlap with the signatures reported by Sivakumaran et al. (*CD3E, PTPRCv1, NOD2*), suggesting that these genes are useful in predicting TB treatment outcome independently of ethnic background. Furthermore, our TB treatment outcome gene signatures showed overlap of genes of the TB risk signature predicting TB progression from healthy controls more than a year before onset of TB (*GBP1, GBP2, GBP5, FCGR1A, STAT1, TAP1*).^22^ Within our study, 12 genes (*BCL2, BMP6, CCL13, CD209, FCGR1A, GBP1, GBP5, INDO, MMP9, MRC1, STAT1, TLR9*) were overlapping between gene signatures, including both the gene signatures obtained by down-sampling and the gene signatures obtained by SMOTE. The occurrence of genes in multiple gene signatures within this study and between studies highlights the power of transcriptomic biomarkers in predicting TB treatment outcome and suggests that universal biomarkers can be applied to cohorts of different ethnicity and independently of the DM/glycaemia status of TB patients.

Patients with a poor TB treatment outcome responded to TB treatment at the level of individual genes, as detected by downregulation of genes (*GBP1, GBP2, GBP5, IFITM3*) that have been associated with active TB and upregulation of genes (*CD3E, PTPRCv1, NLRP1, GNLY, PRF1, BCL2*) that show lower expression in patients with active TB compared to LTBI or healthy controls.^15^^,^^20^^,^^39^^,^^55^ However, MDP analysis showed that the response to TB treatment was diminished in those with a poor TB treatment outcome compared to patients who had a good TB treatment outcome. Notably, the majority of genes that were significantly downregulated in patients who had a good TB treatment outcome, but not in patients who had a poor TB treatment outcome, are involved in IFN signaling (*IRF7, IFIT2, IFIT3, STAT2, IFI6, TAP2*). This suggests that a poor TB treatment outcome was reflected by persisting IFN signaling response and supports a role for type I IFN signaling in TB pathogenesis.^15^^,^^57^

*TAGAP* was significantly increased in patients who had a poor TB treatment outcome in the pooled South African and Indonesian cohort as well as in both cohorts separately. *TAGAP* encodes T-cell activation Rho-GTPase-activating protein, however, the exact role of *TAGAP* in *Mtb* pathogenesis is currently unknown. Several studies have linked *TAGAP* with active TB; *TAGAP* was enriched for differential acetylation peaks upon *Mtb* infection in granulocytes^58^ and *TAGAP* was induced upon vaccination with AERAS-402 vaccine encoding a fusion protein of *Mtb* antigens.^59^ Furthermore, *TAGAP* had higher expression in TB patients compared to LTBI and healthy controls^55^ and, surprisingly, lower expression in pulmonary TB compared to household controls.^60^ Our data showing that *TAGAP* expression was significantly increased during TB treatment in patients who had a poor TB treatment outcome could indicate that *TAGAP* is actively involved in TB pathogenesis or that *TAGAP* expression is a consequence of persisting *Mtb* infection, potentially by enhanced T-cell activation, but this remains to be investigated.

There are several limitations of the current study. First, the sample size in this study was not based on an *a priori* power calculation, as this study was part of a larger study investigating differences in gene expression in patients with varying degrees of hyperglycemia. To increase statistical power, we therefore pooled patients from two cohorts (South Africa and Indonesia), which introduced heterogeneity within the studied groups. However, this can also be a strength, potentially increasing application over different ethnic backgrounds. Second, the low sample size ( = 15) of patients with a poor TB treatment outcome with or without DM (Table 1) reduced the robustness of the identified signatures in a DM-stratified analysis in our prediction model, which is a limitation of this study. TB-DM patients received different DM medications and considering the low sample size, we were unable to correct for this, which could have been a confounding factor. However, we have previously seen similar changed in blood gene expression in people with pre-existing DM taking medication and with transient hyperglycaemia not receiving medication, indicating this has minimal effect.^32^ We have also previously found that metformin has minimal impact on circulating blood transcriptomes,^61^ as do TB drugs.^62^ Our study was also too small to perform a stratified analysis based on the type of poor outcome, *i.e.* death, default, treatment failure or recurrence after treatment completion. We could not determine whether recurrence was due to relapse or reinfection: relapse and failure both occur when insufficient mycobacteria have been killed to permit immunological control. In the future, our model should be tested prospectively in a large TB treatment cohort, to determine its validity for recurrence/relapse as well as failure. Third, there were missing values in the cohort study. The missing values occurred as a result of random drop-outs or technical errors caused by low quantity or quality of some samples, and therefore the use of linear mixed models for the DEA was employed, as it most likely produced unbiased results. Fourth, although the prevalence of hyperglycaemia/DM is not indicated in the majority of other TB biomarker studies, which is a limitation of these studies considering the rising incidence of TB-DM comorbidity, our study contained many patients with high HbAc1 levels. Although this may have introduced a bias, the strength of this approach is that TB treatment outcome signatures have been developed that can be applied to patients independently of their glycaemia/DM status. Furthermore, we showed that our eight-gene diagnosis signature had a high performance (AUC = 0·749) when tested on an external validation cohort in patients with a different ethnic background (India), which is striking since geographic or ethnic variations may significantly impact on the immune responses to TB.

In this study, we demonstrated the potential of gene signatures to predict TB treatment outcome, in a cohort including patients with concomitant DM or hyperglycaemia. Here, we have focused on the host transcriptome, but in later development stages, host and clinical host factors, such as extent of lung cavitation at diagnosis and through treatment, can be added to improve prediction accuracy, as was suggested and demonstrated by Sivakumaran et al.^28^ Identification of a diagnosis gene signature containing only eight genes in this study, and even fewer genes in signatures reported by others,^26^^,^^27^ indicates that clinically-implementable biomarker signatures can be developed using transcriptomic-based approaches using easily accessible whole blood, and that are promising as surrogate marker for sputum culture conversion.

## Contributors

Study concept and design: B.A., R.vC., R.R., P.C.H., G.W., S.A.J., J.A.C., M.C.H., H.M.D., T.H.M.O., J.M.C.; Patient recruitment, Sample collection, processing and selection: B.A., K.R., S.T.M., L.K., P.C.H., K.S., R.R., G.W.; Clinical database design, curation, maintenance: S.K.-B., J.A.C.; Laboratory Experiments and data acquisition: C.E., S.vV., J.S.L., J.M.C.; Data Analysis and interpretation: C.L.R.vD., C.E., S.vV., V.K., S.A.J., C.W., M.C.H., H.M.D., T.H.M.O., E.V., J.M.C.; Writing the manuscript: C.L.R.vD., C.E., S.A.J., H.M.D., T.H.M.O., E.V., J.M.C.; Critical Revision of the manuscript: C.L.R.vD., C.E., K.R., S.T.M., S.A.J., P.C.H., J.A.C., M.C.H., H.M.D., T.H.M.O., E.V., J.M.C.; Data verification: C.L.R.vD., C.E., E.V., J.M.C.; All authors read and approved the final version of the manuscript.

## Data sharing statement

RNA sequence data have been submitted to NCBI Gene Expression Omnibus (GEO) under accession number GSE193979. dcRT-MLPA data can be found in Supplementary Table S6.

## Declaration of interests

G.W. had patents to methods of tuberculosis diagnosis and to tuberculosis biomarkers unrelated to the current study. The rest of the authors declare no financial or commercial conflicts of interest.

## Appendix Supplementary materials

Image, application 1

Image, application 2

Image, application 3

Image, application 4

Image, application 5

Image, application 6

Image, application 7

Image, application 8

Image, application 9

Image, application 10

Image, application 11

Image, application 12

Image, application 13
