# Supplementary material for: Transcriptional profiles predict treatment outcome in patients with tuberculosis and diabetes at diagnosis and at two weeks after initiation of anti-tuberculosis treatment
Source: eBioMedicine. 2022 Jul 15;82:104173. doi: 10.1016/j.ebiom.2022.104173 (PMC9297076; doi:10.1016/j.ebiom.2022.104173)
Supplement: Supplementary file 1 [file mmc1.docx]

Captions for Supplementary Material

**Supplementary Figure S1: Overview of participant enrolment in the TANDEM study, classification and inclusion in the longitudinal gene expression analysis.**Dx: diagnosis; M: month; W: week.

**Supplementary Figure S2. MDP plot representing the change in gene expression perturbation in TB patients from South Africa categorized based on treatment outcome.** Blood transcriptomes from TB patients who had a good or poor treatment outcome were determined by dcRT-MLPA. The extent of overall difference in gene expression, relative to the median of expression in healthy controls, was calculated for individual patients at the timepoints shown. The bars and whiskers show the median and data within the Q1-1·5 x inter quartile range (IQR) and Q3+1·5 x IQR interval. Differences were significant by Mann-Whitney U-test with Benjamini-Hochberg correction for multiple testing.

* p<0·05, ** p<0·01, *** p<0·001, **** p<0·0001.

**Supplementary Figure S3. Differential change in gene expression in TB patients through treatment in those who had a good or poor treatment outcome.** MaSigPro analysis was conducted on the blood RNA-Seq data from TB patients from South Africa (a) or Indonesia (b), to identify genes which were significantly differentially expressed between those patients with a good or poor outcome. Plots show hierarchical clusters of genes, and bars show mean ± 1 SEM. Data were filtered to remove lowly abundant transcripts prior to analysis.

**Supplementary Figure S4. Cell population estimates in good and poor treatment outcomes in South Africa and Indonesia.** Estimates of relative differences in cell proportions were calculated from RNA-seq data using R package CellCODE. IRIS and DMAP data sets used as reference. Bars and whiskers show median and 1·5 x IQR. There were no significant differences identified between Good and Poor TB treatment outcome groups at any timepoint for any cell type.

**Supplementary Figure S5. Differential expression analysis in patients from South Africa and Indonesia who had a poor treatment outcome versus patients who had a good treatment outcome at the indicated timepoints.** Non-parametric Mann-Whitney U-test with Benjamini-Hochberg correction for multiple testing was applied to test for statistical differences between the groups.

**Supplementary Figure S6. Differential expression analysis of all TB patients from the South African and Indonesian cohorts categorized based on treatment outcome compared to their gene expression levels at diagnosis.** (a) Volcano plots representing DEGs regulated during TB treatment of TB patients from the South African cohort who had a good treatment outcome (left panel) or a poor treatment outcome (right panel). (b) Volcano plots representing DEGs regulated during TB treatment of TB patients from the Indonesian cohort who had a good treatment outcome (left panel) or a poor treatment outcome (right panel). (a,b) The y-axis scales of the plots are harmonized per treatment outcome. -log10-transformed p-values are plotted against log2 FC. Genes with p <0·05 and log2 FC <-0·6 or >0·6 were labelled as DEGs.

**Supplementary Figure S7. Scatter plot representing Pearson correlations between the longitudinal DEGs in TB patients who had a poor treatment outcome versus the longitudinal DEGs in TB patients who had a good treatment outcome.** Values are plotted as log2 FC (month 6-diagnosis). Black line corresponds to line of best fit and shaded bands indicate confidence intervals. Red shaded areas indicate genes that were identified as DEGs only in patients who had a good treatment outcome and blue shaded areas indicate genes that were identified as DEGs only in patients who had a poor treatment outcome.

**Supplementary Figure S8. Scatter plots representing Pearson correlations between the longitudinal DEGs identified by dcRT-MLPA versus the same genes identified by RNA-Seq.** Values are plotted as log2 FC (month six-diagnosis). Black line corresponds to line of best fit and shaded bands indicate confidence intervals.

**Supplementary Figure S9. Prediction of treatment outcome in RNA-Seq data from peripheral blood.** ROC curves showing the predictive power of (a) the gene signatures identified in the pooled cohort (South Africa and Indonesia) or (b) the Sweeney gene signature to classify TB patients at the indicated timepoints after initiation of TB treatment into patients who had a good treatment outcome and patients who had a poor treatment outcome. Data were split into training and test sets (60/40). For each time point a gene signature was generated by RFE and a weighted model fitted using glmnet method. Weights of 1/frequency * 0.5 were used. Abbreviations: AUC, area under the curve; CI, confidence interval.

**Supplementary Figure S10. Performance evaluation of the gene signatures identified without discriminating between diabetes (DM) conditions in predicting the outcome in TB treatment in patients with TB and DM.** ROC curves showing the predictive power of the gene signatures identified in the balanced pooled cohort without discriminating between DM conditions to classify TB patients with DM into patients who had a good treatment outcome and patients who had a poor treatment outcome, using RFE – RF model and LOOCV. The dataset was balanced by down-sampling to encompass the same number of individuals with poor and good treatment outcome. Abbreviations: AUC, area under the curve; CI, confidence interval.

**Supplementary Figure S11. PCA analysis of Sex of TB patients in South Africa and Indonesia.** TB patients are classified into good TB treatment outcome (left panel) and poor TB treatment outcome (right panel) at diagnosis. Abbreviations: PC, principal component.

**Supplementary Figure S12. PCA analysis of Age of TB patients in South Africa and Indonesia.** Plots showing (a) PC2 versus PC1 or (b) the correlation between Age and PC1 in TB patients that are classified into good TB treatment outcome (left panels) and poor TB treatment outcome (right panels) at diagnosis. Abbreviations: PC, principal component.

**Supplementary Figure S13. PCA analysis of BMI of TB patients in South Africa and Indonesia.** Plots showing (a) PC2 versus PC1 or (b) the correlation between BMI and PC1 in TB patients that are classified into good TB treatment outcome (left panels) and poor TB treatment outcome (right panels) at diagnosis. Abbreviations: PC, principal component.

**Supplementary Figure S14. PCA analysis of HbA1c levels at diagnosis of TB patients in South Africa and Indonesia.** Plots showing (a) PC2 versus PC1 or (b) the correlation between HbA1c and PC1 in TB patients that are classified into good TB treatment outcome (left panels) and poor TB treatment outcome (right panels) at diagnosis. Abbreviations: PC, principal component.

**Supplementary Figure S15. Identification of a delta (week two minus diagnosis) signature predicting the outcome of TB treatment.** ROC curve showing the predictive power of the gene signature identified in the balanced pooled cohort to classify TB patients into patients who had a good treatment outcome and patients who had a poor treatment outcome, using the RFE - RF model and LOOCV. The dataset was balanced by down-sampling to encompass the same number of individuals with poor and good treatment outcome. Abbreviations: AUC, area under the curve; CI, confidence interval.

**Supplementary Figure S16. Venn diagrams showing the number of genes encompassing treatment outcome signa-tures as identified by RFE-RF models.** (a) Venn diagram displaying the number of unique and overlapping genes comparing diagnosis (blue), week two (orange) and month two (green) gene signatures obtained by random-downsampling. (b) Venn diagram displaying the number of unique and overlapping genes comparing the gene signatures in the current study (diagnosis, week two and month two; orange) obtained by random-downsampling with the gene signatures published by Sivakumaran et al. (blue). (c) Venn diagram displaying the number of unique and overlapping genes comparing the diagnosis gene signature obtained by random-downsampling (blue) with the diagnosis gene signature obtained by applying SMOTE sampling (orange) to balance the classes. Overlapping genes are annotated.

**Supplementary Figure S17. Identification of a SMOTE diagnosis signature predicting the outcome of TB treatment.** ROC curves showing the predictive power of the gene signatures identified in the balanced pooled cohort (South Africa and Indonesia) and validated in the South African and Indonesian cohort or in an external Indian cohort. TB patients are classified into patients who had a good treatment outcome and patients who had a poor treatment outcome at diagnosis, using the RFE - RF model and LOOCV. The dataset was balanced by SMOTE to encompass the same number of individuals with poor and good treatment outcome. Abbreviations: AUC, area under the curve; CI, confidence interval.
