# Supplementary material for: Transcriptional profiles predict treatment outcome in patients with tuberculosis and diabetes at diagnosis and at two weeks after initiation of anti-tuberculosis treatment
Source: eBioMedicine. 2022 Jul 15;82:104173. doi: 10.1016/j.ebiom.2022.104173 (PMC9297076; doi:10.1016/j.ebiom.2022.104173)
Supplement: Supplementary file 2 [file mmc2.pdf]

Supplementary Information

Reverse-Transcriptase Multiplex Ligation-dependent Probe Amplification (dcRT-MLPA)

For each target-specific sequence, a specific RT primer was designed located immediately downstream of the left- and right-hand half-probe target sequence. 125 ng RNA was reverse transcribed to cDNA by incubation at 37°C for 15 min, using RT-primer mix and Moloney Murine Leukemia Virus (M-MLV) reverse transcriptase (Promega, Leiden, The Netherlands). Reverse transcriptase was inactivated by heating at 98ºC for two minutes. The left- and right-hand half probes were hybridized to the cDNA at 60ºC overnight and annealed half-probes were ligated at 54ºC for 15 minutes using ligase-65 (MRC-Holland). Ligase-65 was subsequently inactivated by heating at 98ºC for five minutes. Ligated probes were amplified by PCR: 33 cycles at 95ºC for 30 seconds, 58ºC for 30 seconds and 72ºC for 60 seconds, followed by one cycle at 72ºC for 20 minutes. PCR products were 1:10 diluted in Highly deionized (Hi-Di) formamide (ThermoFisher) containing 400HD Rhodamine X (ROX) fluorophore size standard (ThermoFisher). PCR products were denatured at 95ºC for five minutes, stored immediately at 4ºC and analyzed on an Applied Biosystems 3730 capillary sequencer in GeneScan mode (BaseClear, Leiden, The Netherlands). Trace data were analyzed using GeneMapper software 5 (Applied Biosystems, Warrington, UK). The areas of each assigned peak (arbitrary units) were exported for analysis in R (version 3.6.3). Data were corrected for batch effect and normalized to housekeeping gene glyceraldehyde 3-phosphate dehydrogenase (*GAPDH*). Signals below the threshold value for noise cutoff in GeneMapper (log2 transformed peak area 7·64) were assigned the threshold value for noise cutoff.

RT primers and half-probes were designed by Leiden University Medical Centre (LUMC, Leiden, The Netherlands) and comprised sequences for four housekeeping genes and 144 selected key immune-related genes to profile the following compartments of the human

immune response (Supplementary Table S1): (1) Adaptive immune responses: T-cell responses; Th1 responses; Th2 responses; Th17/22 responses; Treg responses; T-cell cytotoxicity; Immune cell subset markers including B-cells and NK-cells. (2) Innate immune responses: Myeloid-associated markers and scavenger receptors; Pattern recognition receptors; Inflammasome components. (3) Inflammatory and IFN-signalling genes. (4) Other genes: Anti-microbial activity; Apoptosis/cell survival; E3 ubiquitin protein ligases; Small GTPases/(Rho)GTPase activating proteins; Additional chemokines; Cell growth/proliferation; Cell activation; Transcriptional regulators/activators; Intracellular transport; Mitochondrial Stress/Proteasome; Inflammation.

Linear Mixed Models for identification of DEGs

Longitudinal DEGs were identified by means of linear mixed models using the lmer function of the lme4 package in R (1). To increase statistical power, datasets of all TB patients included in the South African and Indonesian cohorts were pooled independent of diabetes/glycaemia status and split based on TB treatment outcome. Models were fitted on *GAPDH*-normalized log_2-transformed targeted gene expression data. Outcome-time interactions were included as fixed effects and the patients ID-time interactions were included as random effects.

Identification of gene signatures for TB treatment outcome

For modelling analyses, RNA-Seq data were randomly split into training and test sets (60/40) using the R package *caret* (2). Feature selection was performed for each timepoint using RFE (3) with repeated cross validation as the re-sampling method (n=10). A weighted model was fitted using glmnet method, using weights 1/frequency * 0·5 and repeated cross validation for re-sampling (n=10). Each model was used to make predictions on the corresponding test set.

To identify signatures associated with TB treatment outcome in dcRT-MLPA data, TB patients of South Africa and Indonesia were pooled independent of diabetes/glycaemia status. To balance the dataset, we applied two random sampling approaches using R: (1) a down-sampling approach, reducing the number of patients in the majority class (i.e. good TB treatment outcome) and (2) an up-sampling approach by generating synthetic data from existing data using the SMOTE (4) function from the *DMwR* package in R, resulting in equal numbers of patients in both classes. Then, RFE (3), available in the *caret* R package (2), was applied with K-fold validation (K=10) to the entire data set search for the optimal combination and number of top-ranking genes able to separate TB patients with a good and poor TB treatment outcome. RFE is a powerful approach for variable selection in high-dimensional data by selecting features that fit a model and removing the weakest feature (or features) until the specified number of features is reached. Once the best predictors of TB treatment outcome were identified, the expression values of these genes were extracted from the dataset. We subsequently applied RF (5) as machine learning algorithm on the dataset and evaluated the performance by LOOCV, both available on the *caret* R package (2).

References

1. Bates D, Mächler M, Bolker B, Walker S. Fitting linear mixed-effects models using lme4. arXiv preprint arXiv:14065823. 2014.
2. Kuhn M. Building predictive models in R using the caret package. Journal of statistical software. 2008;28(1):1-26.
3. Gregorutti B, Michel B, Saint-Pierre P. Correlation and variable importance in random forests. Statistics and Computing. 2017;27(3):659-78.
4. Chawla NV, Bowyer KW, Hall LO, Kegelmeyer WP. SMOTE: synthetic minority over-sampling technique. Journal of artificial intelligence research. 2002;16:321-57.
5. Liaw A, Wiener M. Classification and regression by randomForest. R news. 2002;2(3):18-22.

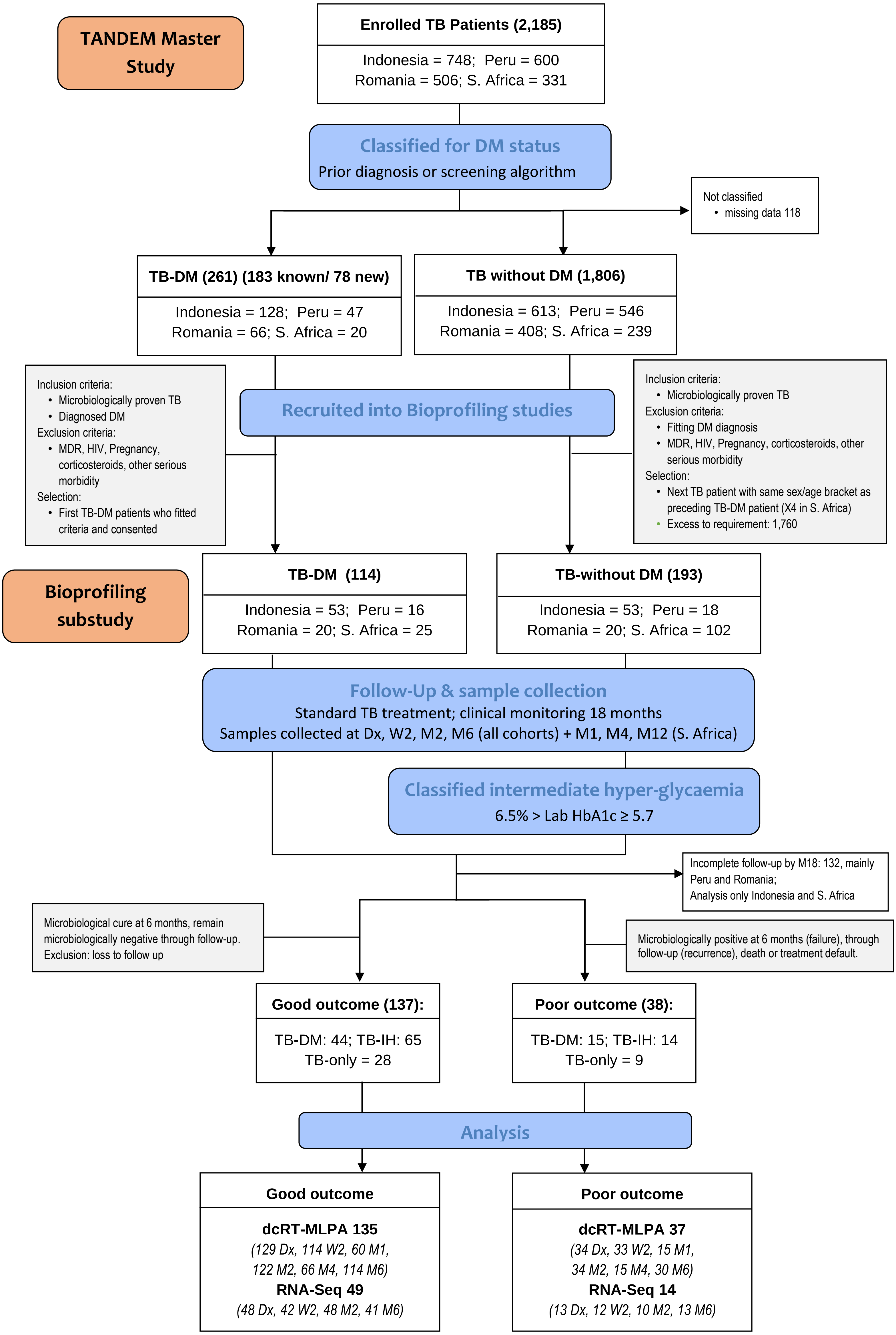

TANDEM Master Study
Enrolled TB Patients (2,185)
Indonesia = 748; Peru = 600
Romania = 506; S. Africa = 331
Classified for DM status
Prior diagnosis or screening algorithm
Not classified
• missing data 118
TB-DM (261) (183 known/ 78 new)
Indonesia = 128; Peru = 47
Romania = 66; S. Africa = 20
TB without DM (1,806)
Indonesia = 613; Peru = 546
Romania = 408; S. Africa = 239
Inclusion criteria:
• Microbiologically proven TB
• Diagnosed DM
Exclusion criteria:
• MDR, HIV, Pregnancy, corticosteroids, other serious morbidity
Selection:
• First TB-DM patients who fitted criteria and consented
Recruited into Bioprofiling studies
Inclusion criteria:
• Microbiologically proven TB
Exclusion criteria:
• Fitting DM diagnosis
• MDR, HIV, Pregnancy, corticosteroids, other serious morbidity
Selection:
• Next TB patient with same sex/age bracket as preceding TB-DM patient (X4 in S. Africa)
• Excess to requirement: 1,760
Bioprofiling substudy
TB-DM (114)
Indonesia = 53; Peru = 16
Romania = 20; S. Africa = 25
TB-without DM (193)
Indonesia = 53; Peru = 18
Romania = 20; S. Africa = 102
Follow-Up & sample collection
Standard TB treatment; clinical monitoring 18 months
Samples collected at Dx, W2, M2, M6 (all cohorts) + M1, M4, M12 (S. Africa)
Classified intermediate hyper-glycaemia
6.5% > Lab HbA1c ≥ 5.7
Incomplete follow-up by M18: 132, mainly Peru and Romania;
Analysis only Indonesia and S. Africa
Microbiological cure at 6 months, remain microbiologically negative through follow-up.
Exclusion: loss to follow up
Microbiologically positive at 6 months (failure), through follow-up (recurrence), death or treatment default.
Good outcome (137):
TB-DM: 44; TB-IH: 65
TB-only = 28
Poor outcome (38):
TB-DM: 15; TB-IH: 14
TB-only = 9
Analysis
Good outcome
dcRT-MLPA 135
(129 Dx, 114 W2, 60 M1,
122 M2, 66 M4, 114 M6)
RNA-Seq 49
(48 Dx, 42 W2, 48 M2, 41 M6)
Poor outcome
dcRT-MLPA 37
(34 Dx, 33 W2, 15 M1,
34 M2, 15 M4, 30 M6)
RNA-Seq 14
(13 Dx, 12 W2, 10 M2, 13 M6)

Supplementary Figure S1: Overview of participant enrolment in the TANDEM study, classification and inclusion in the longitudinal gene expression analysis. Dx: diagnosis; M: month; W: week.

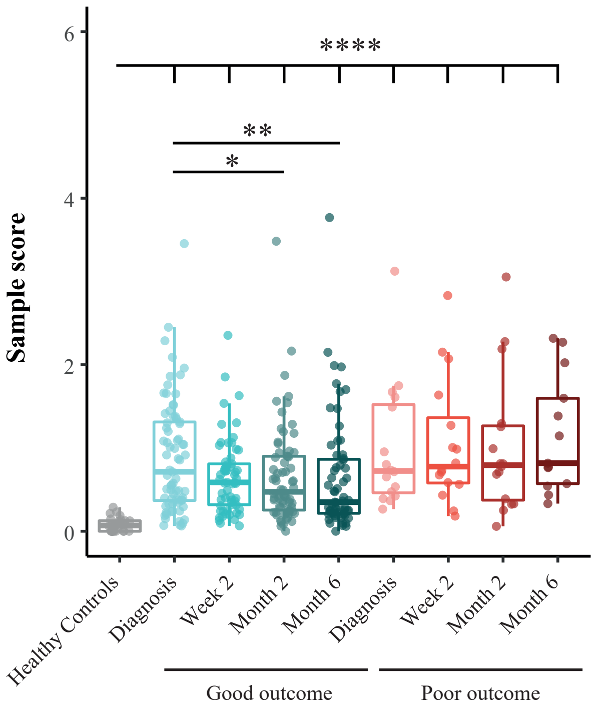

Supplementary Figure S2. MDP plot representing the change in gene expression perturbation in TB patients from South Africa categorized based on treatment outcome. Blood transcriptomes from TB patients who had a good or poor treatment outcome were determined by dcRT-MLPA. The extent of overall difference in gene expression, relative to the median of expression in healthy controls, was calculated for individual patients at the timepoints shown. The bars and whiskers show the median and data within the Q1-1·5 x inter quartile range (IQR) and Q3+1·5 x IQR interval. Differences were significant by Mann-Whitney U-test with Benjamini-Hochberg correction for multiple testing.
* $p<0{\cdot}05$, ** $p<0{\cdot}01$, *** $p<0{\cdot}001$, **** $p<0{\cdot}0001$.

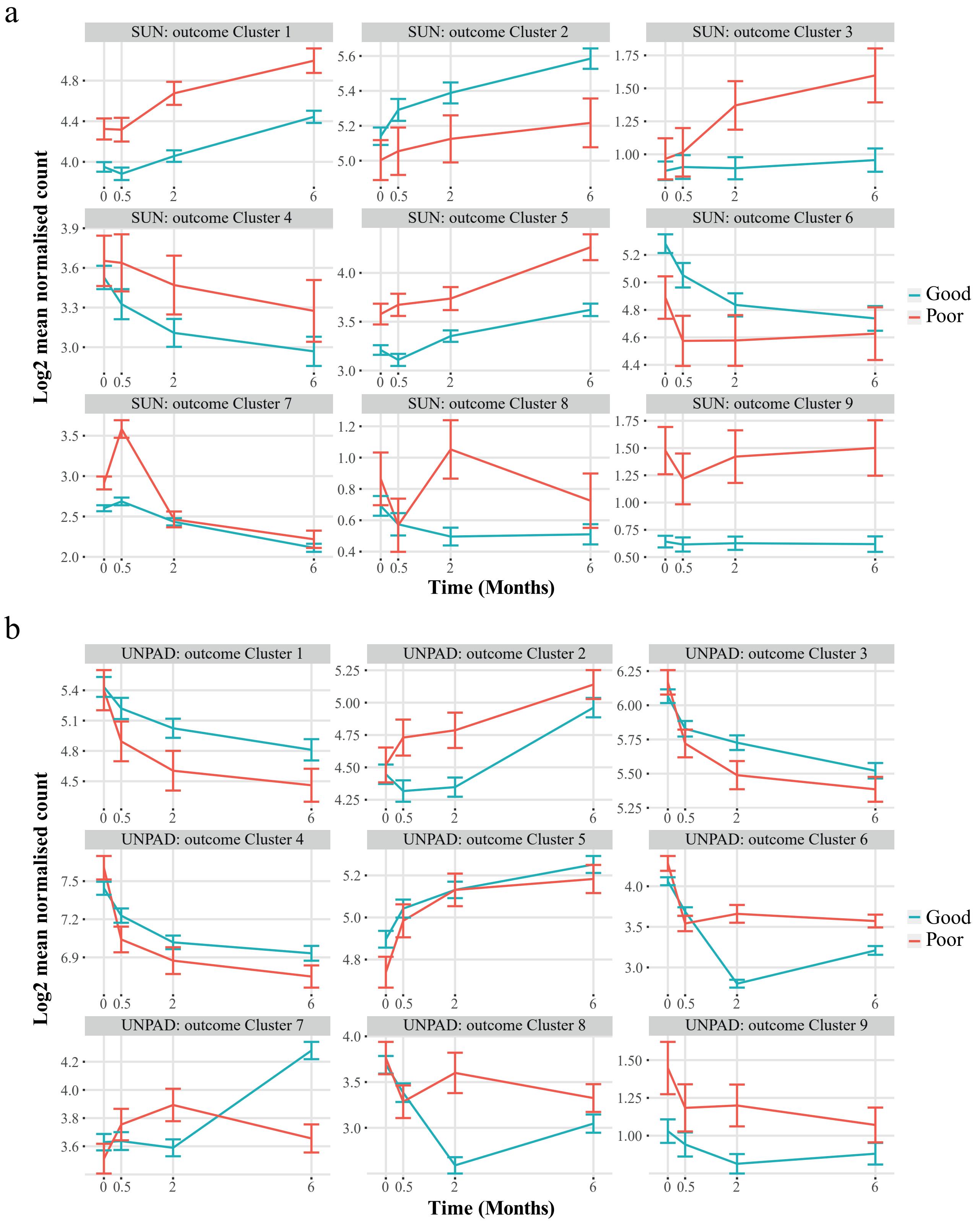

Supplementary Figure S3. Differential change in gene expression in TB patients through treatment in those who had a good or poor treatment outcome. MaSigPro analysis was conducted on the blood RNA-Seq data from TB patients from South Africa (a) or Indonesia (b), to identify genes which were significantly differentially expressed between those patients with a good or poor outcome. Plots show hierarchical clusters of genes, and bars show mean ± 1 SEM. Data were filtered to remove lowly abundant transcripts prior to analysis.

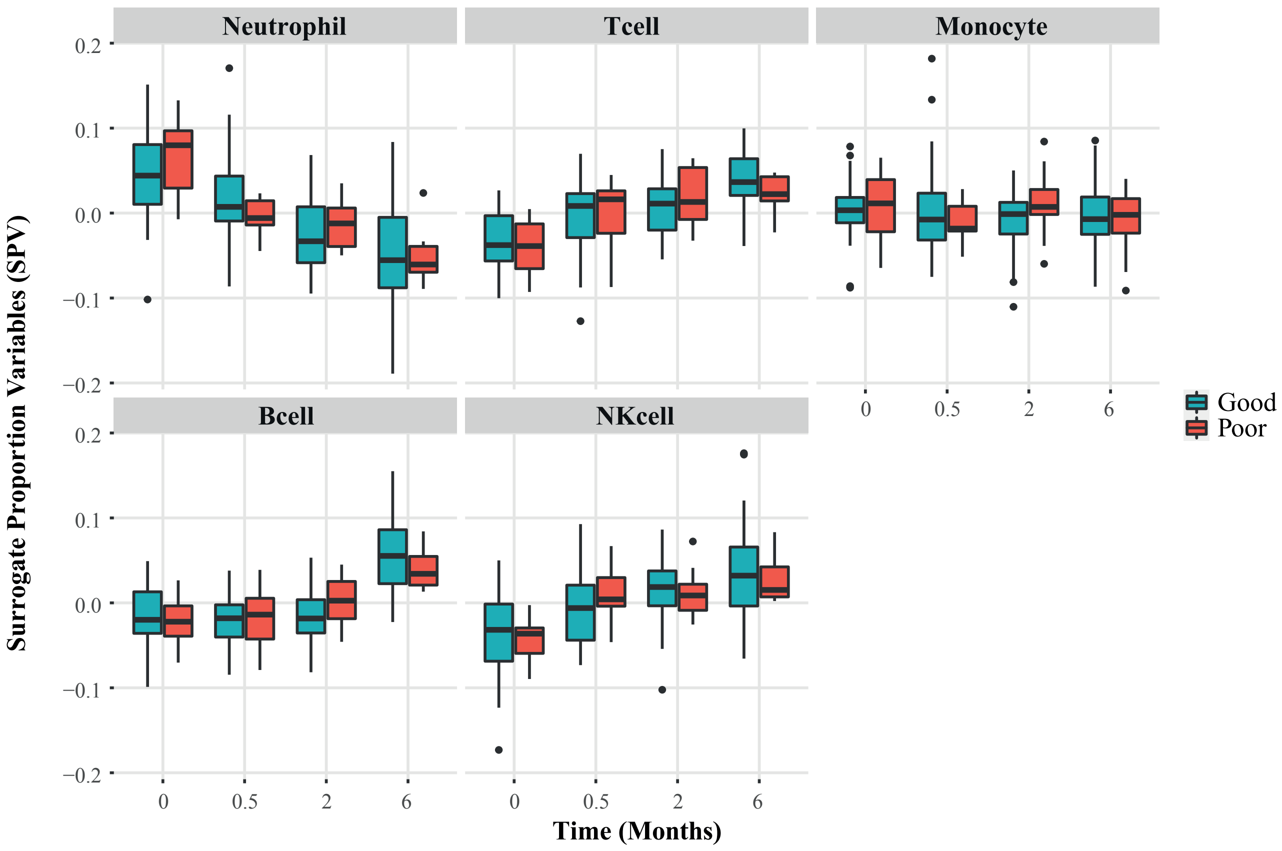

Supplementary Figure S4. Cell population estimates in good and poor treatment outcomes in South Africa and Indonesia. Estimates of relative differences in cell proportions were calculated from RNA-seq data using R package CellCODE. IRIS and DMAP data sets used as reference. Bars and whiskers show median and 1·5 x IQR. There were no significant differences identified between Good and Poor TB treatment outcome groups at any timepoint for any cell type.

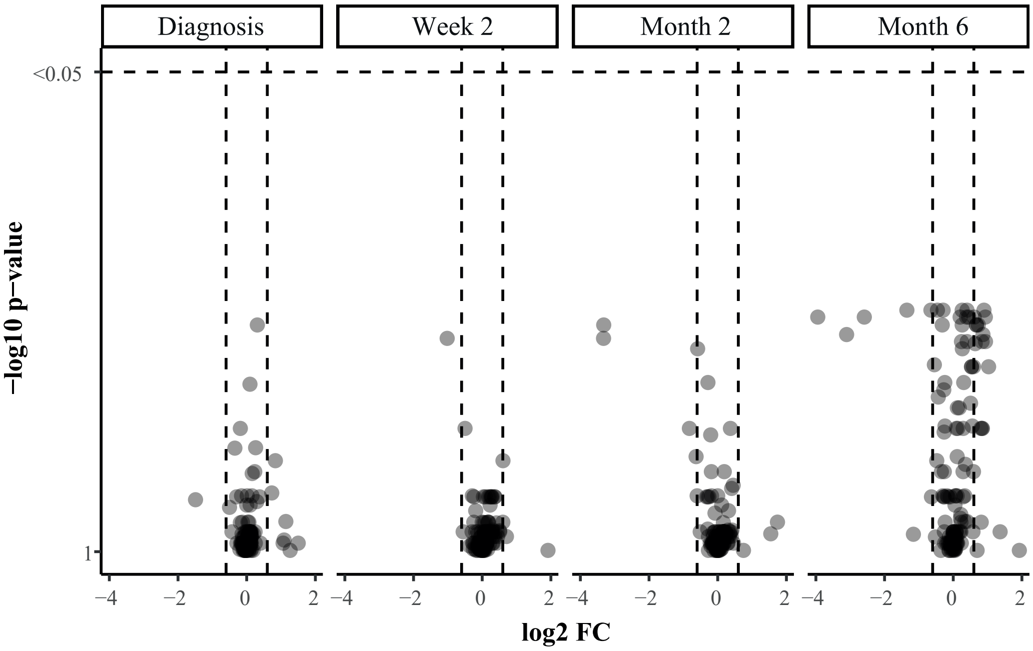

Supplementary Figure S5. Differential expression analysis in patients from South Africa and Indonesia who had a poor treatment outcome versus patients who had a good treatment outcome at the indicated timepoints. Non-parametric Mann-Whitney U-test with Benjamini-Hochberg correction for multiple testing was applied to test for statistical differences between the groups.

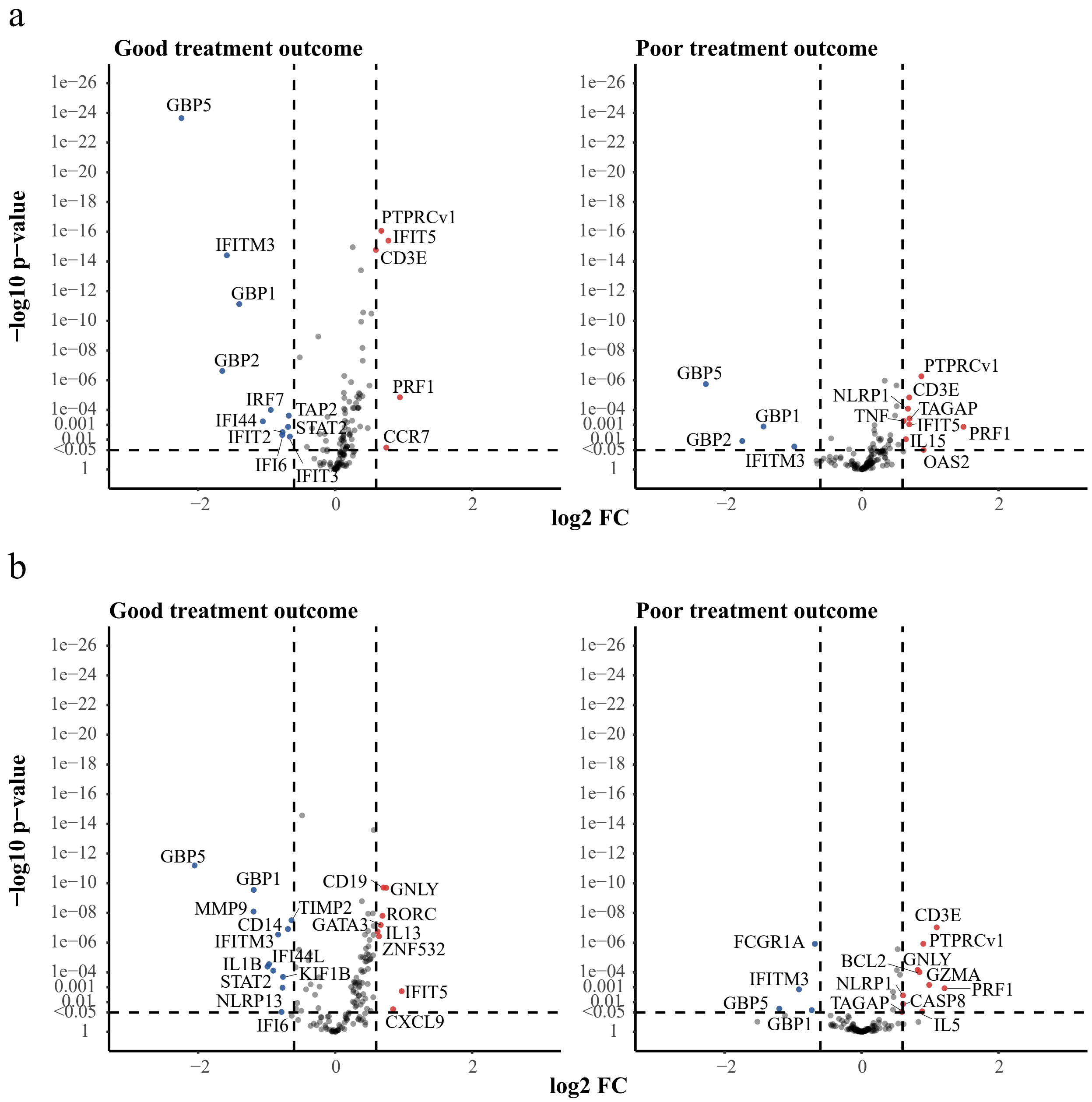

Supplementary Figure S6. Differential expression analysis of all TB patients from the South African and Indonesian cohorts categorized based on treatment outcome compared to their gene expression levels at diagnosis. (a) Volcano plots representing DEGs regulated during TB treatment of TB patients from the South African cohort who had a good treatment outcome (left panel) or a poor treatment outcome (right panel). (b) Volcano plots representing DEGs regulated during TB treatment of TB patients from the Indonesian cohort who had a good treatment outcome (left panel) or a poor treatment outcome (right panel). (a,b) The y-axis scales of the plots are harmonized per treatment outcome. -log10-trans-formed p-values are plotted against log2 FC. Genes with p <0·05 and log2 FC <-0·6 or >0·6 were labelled as DEGs.

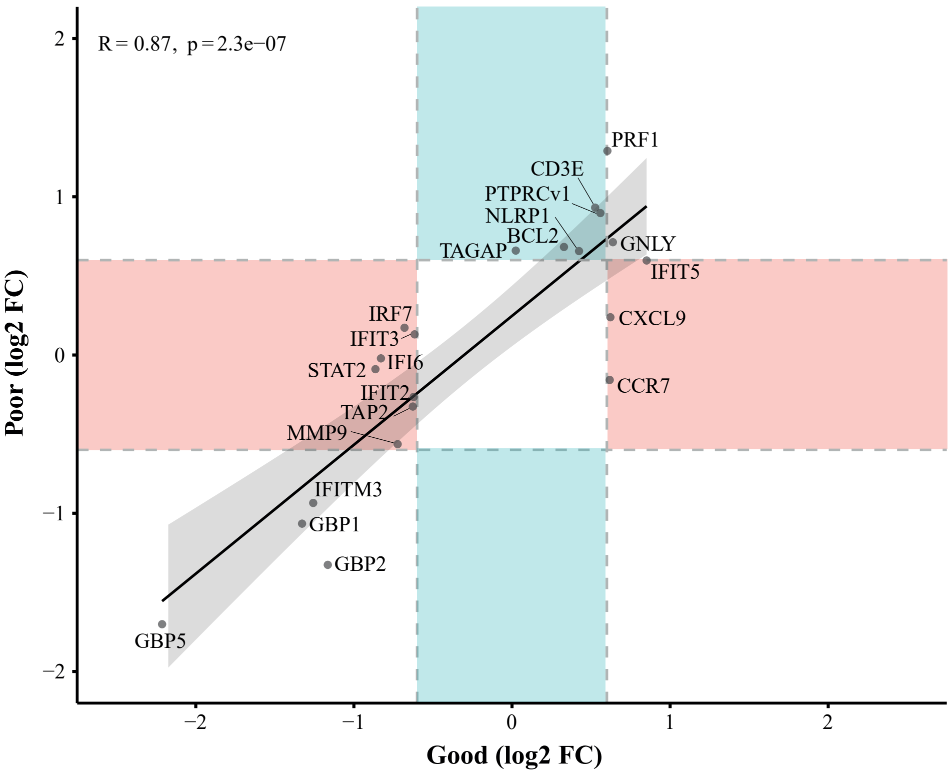

Supplementary Figure S7. Scatter plot representing Pearson correlations between the longitudinal DEGs in TB patients who had a poor treatment outcome versus the longitudinal DEGs in TB patients who had a good treatment outcome. Values are plotted as log2 FC (month 6-diagnosis). Black line corresponds to line of best fit and shaded bands indicate confidence intervals. Red shaded areas indicate genes that were identified as DEGs only in patients who had a good treatment outcome and blue shaded areas indicate genes that were identified as DEGs only in patients who had a poor treatment outcome.

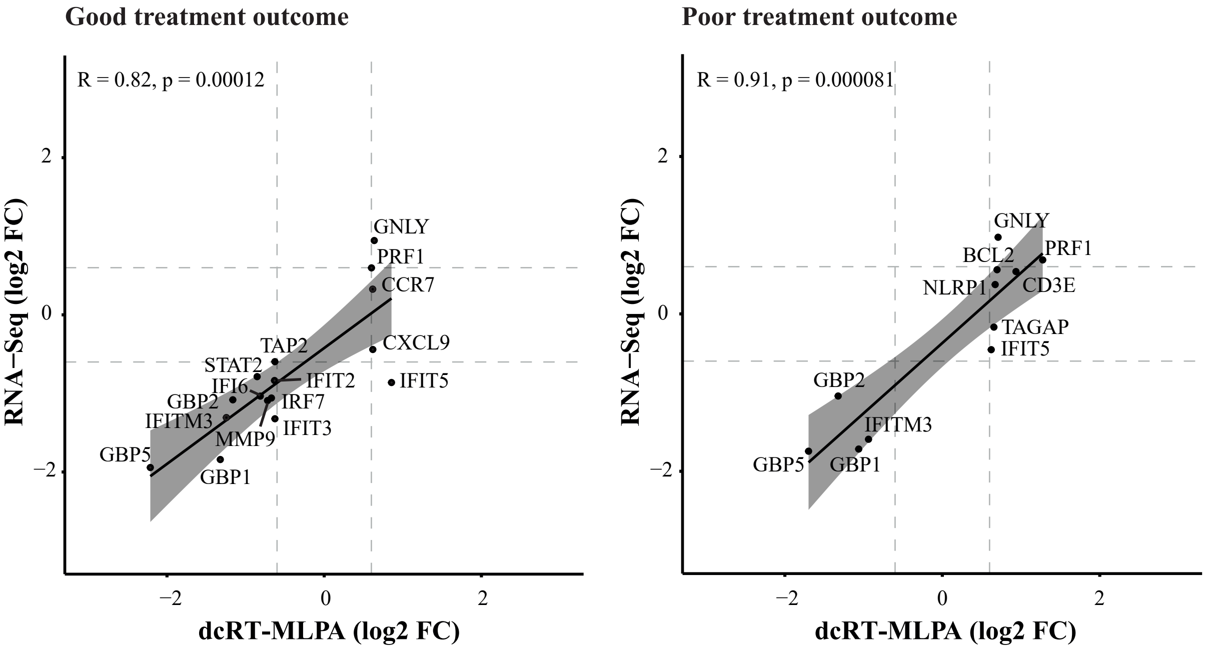

Supplementary Figure S8. Scatter plots representing Pearson correlations between the longitudinal DEGs identified by dcRT-MLPA versus the same genes identified by RNA-Seq. Values are plotted as log2 FC (month six-diagnosis). Black line corresponds to line of best fit and shaded bands indicate confidence intervals.

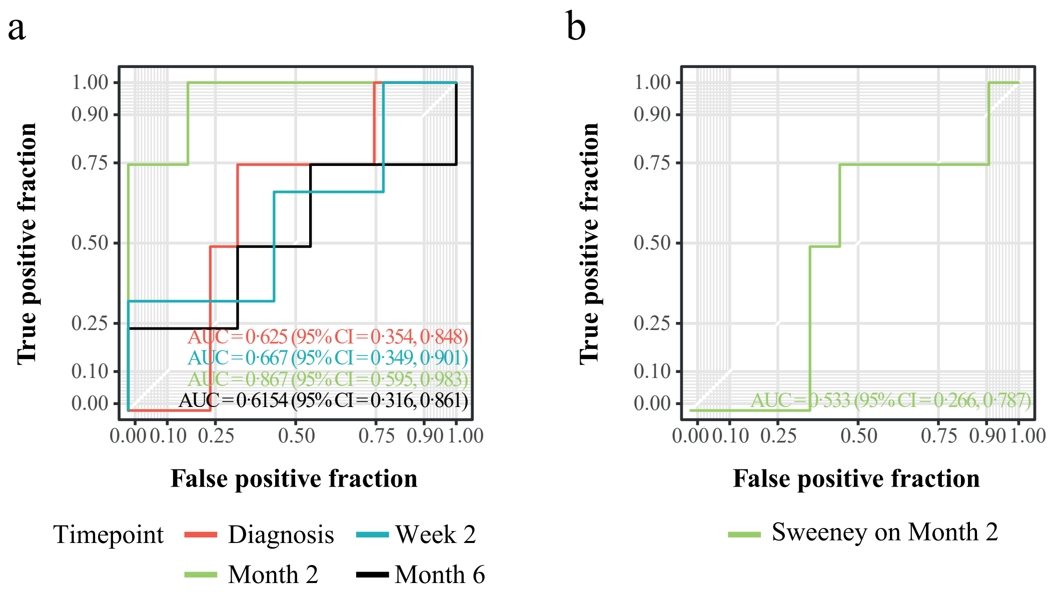

Supplementary Figure S9. Prediction of treatment outcome in RNA-Seq data from peripheral blood. ROC curves showing the predictive power of (a) the gene signatures identified in the pooled cohort (South Africa and Indonesia) or (b) the Sweeney gene signature to classify TB patients at the indicated timepoints after initiation of TB treatment into patients who had a good treatment outcome and patients who had a poor treatment outcome. Data were split into training and test sets (60/40). For each time point a gene signature was generated by RFE and a weighted model fitted using glmnet method. Weights of 1/frequency * 0.5 were used. Abbreviations: AUC, area under the curve; CI, confidence interval.

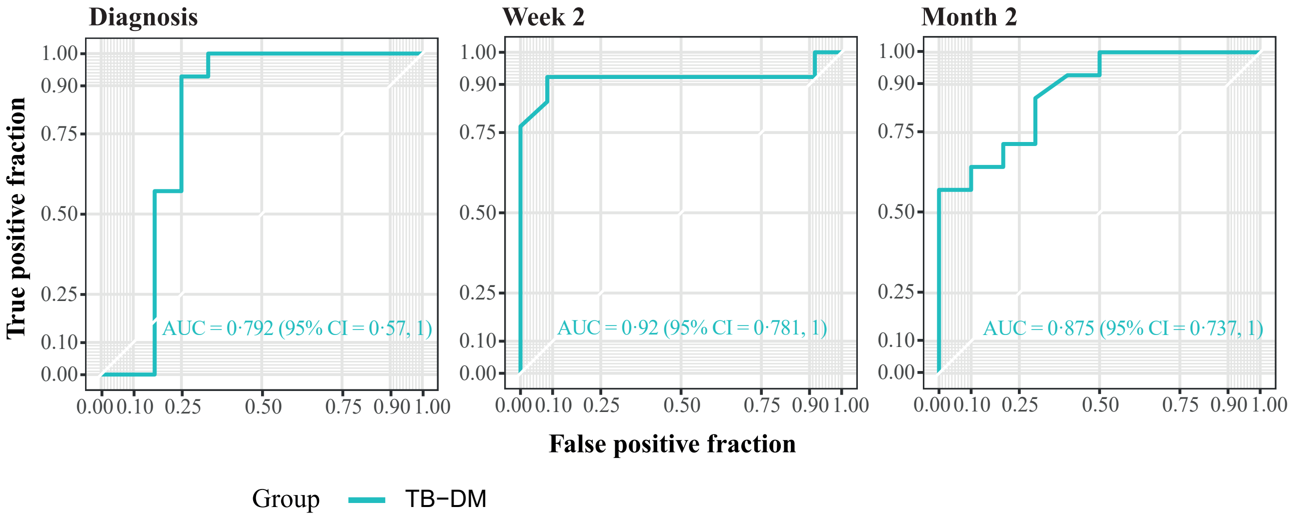

Supplementary Figure S10. Performance evaluation of the gene signatures identified without discriminating between DM conditions in predicting the outcome of TB treatment in participants with TB and diabetes. ROC curve showing the predictive power of the gene signature identified in the balanced pooled cohort without discriminating between DM conditions to classify TB patients with diabetes into patients who had a good treatment outcome and patients who had a poor treatment outcome, using the RFE - RF model and LOOCV. The dataset was balanced by down-sampling to encompass the same number of individuals with poor and good treatment outcome. Abbreviations: AUC, area under the curve; CI, confidence interval.

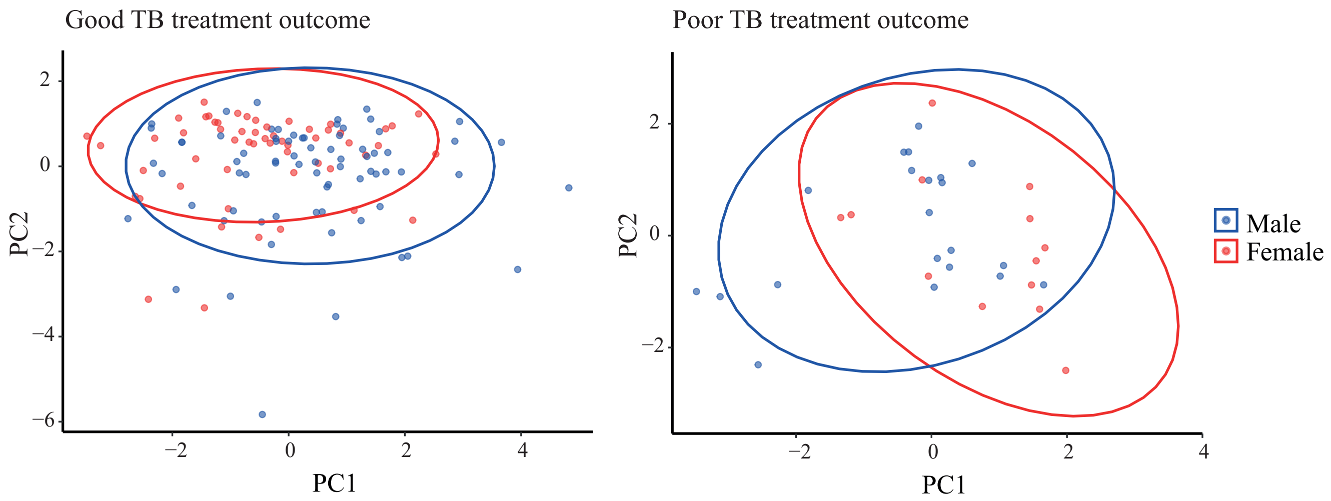

Supplementary Figure S11. PCA analysis of Sex of TB patients in South Africa and Indonesia. TB patients are classified into good TB treatment outcome (left panel) and poor TB treatment outcome (right panel) at diagnosis. Abbreviations: PC, principal component.

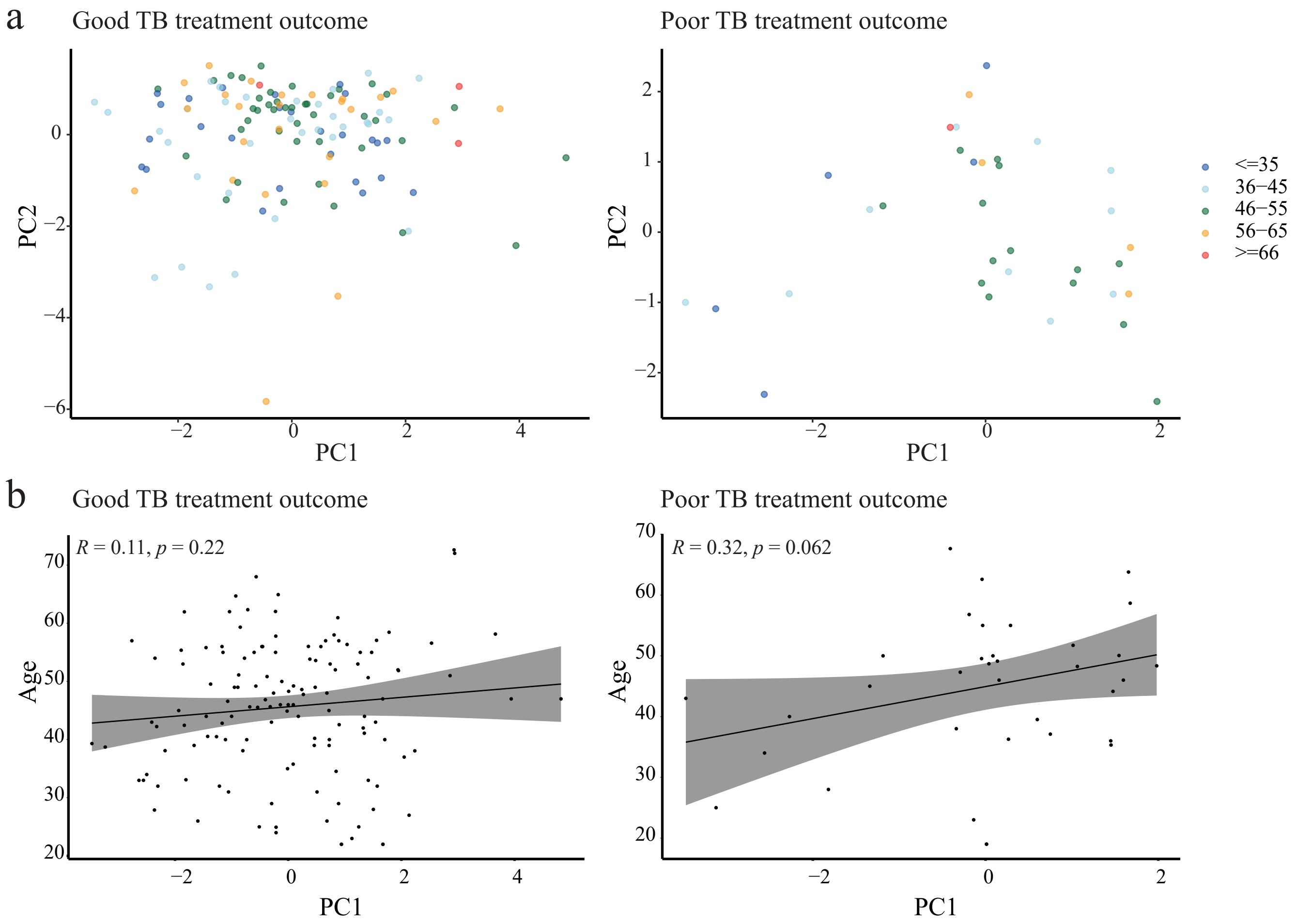

Supplementary Figure S12. PCA analysis of Age of TB patients in South Africa and Indonesia. Plots showing (a) PC2 versus PC1 or (b) the correlation between Age and PC1 in TB patients that are classified into good TB treatment outcome (left panels) and poor TB treatment outcome (right panels) at diagnosis. Abbreviations: PC, principal component.

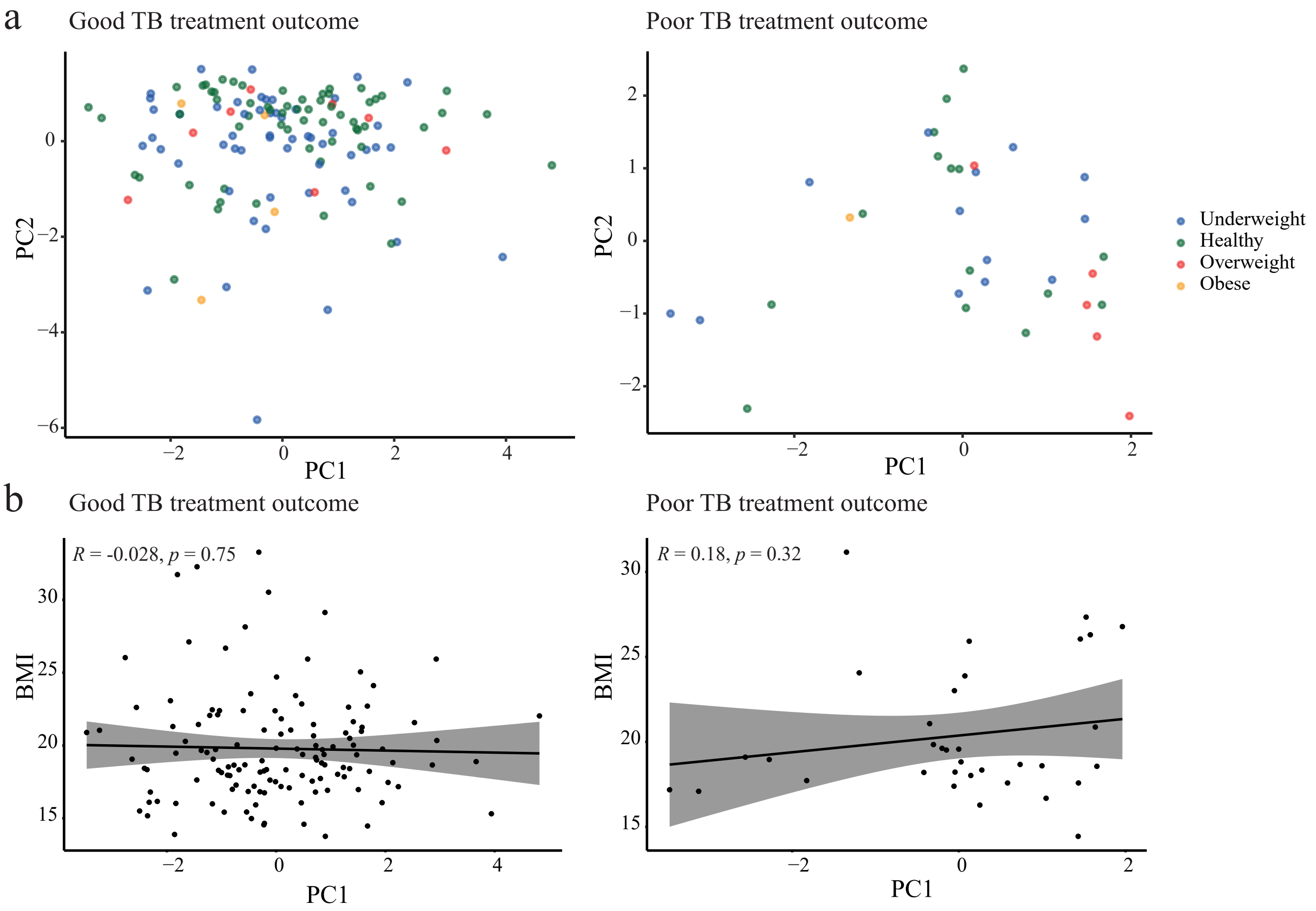

Supplementary Figure S13. PCA analysis of BMI of TB patients in South Africa and Indonesia. Plots showing (a) PC2 versus PC1 or (b) the correlation between BMI and PC1 in TB patients that are classified into good TB treatment outcome (left panels) and poor TB treatment outcome (right panels) at diagnosis. Abbreviations: PC, principal component.

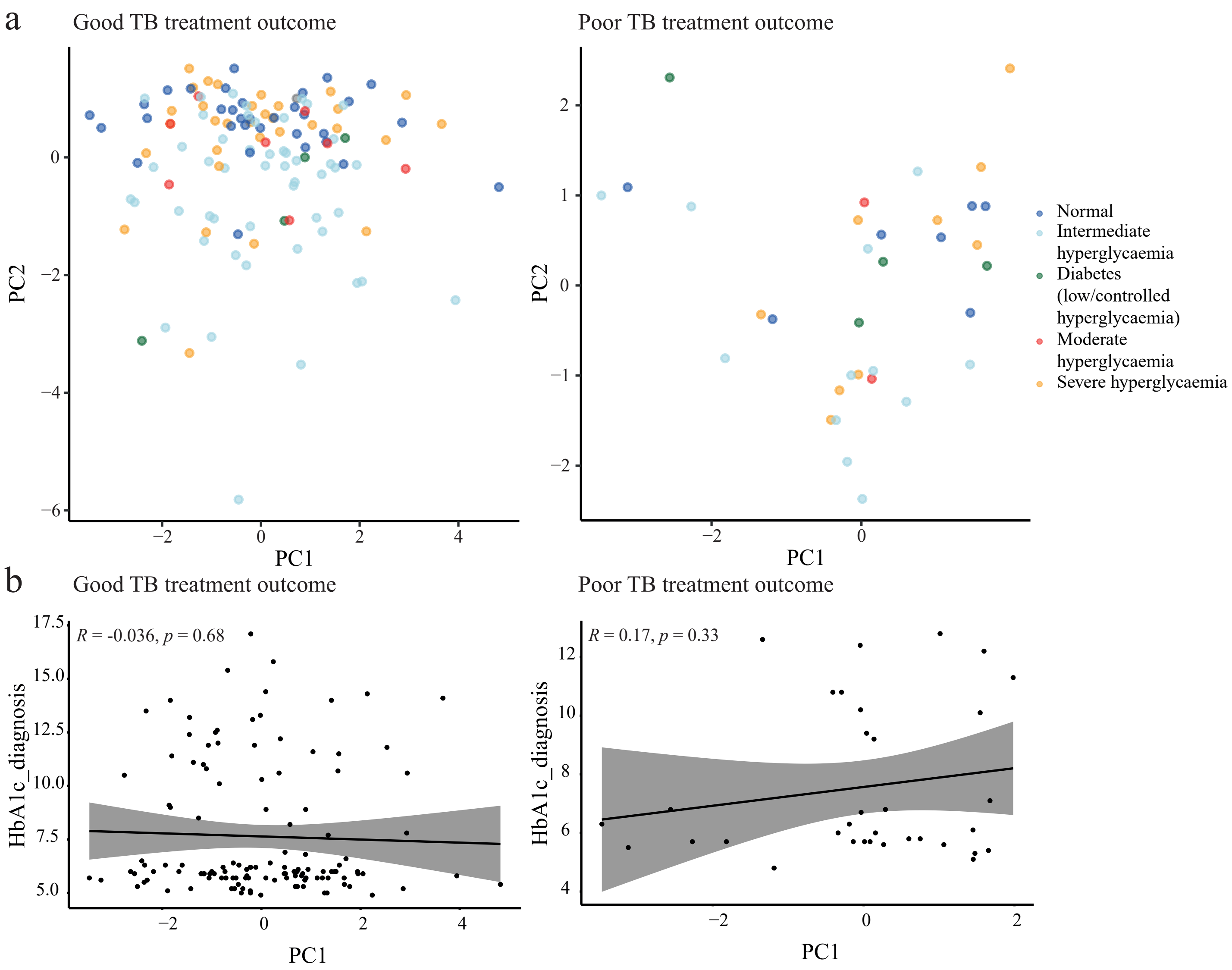

Supplementary Figure S14. PCA analysis of HbA1c levels at diagnosis of TB patients in South Africa and Indonesia. Plots showing (a) PC2 versus PC1 or (b) the correlation between HbA1c and PC1 in TB patients that are classified into good TB treatment outcome (left panels) and poor TB treatment outcome (right panels) at diagnosis. Abbreviations: PC, principal

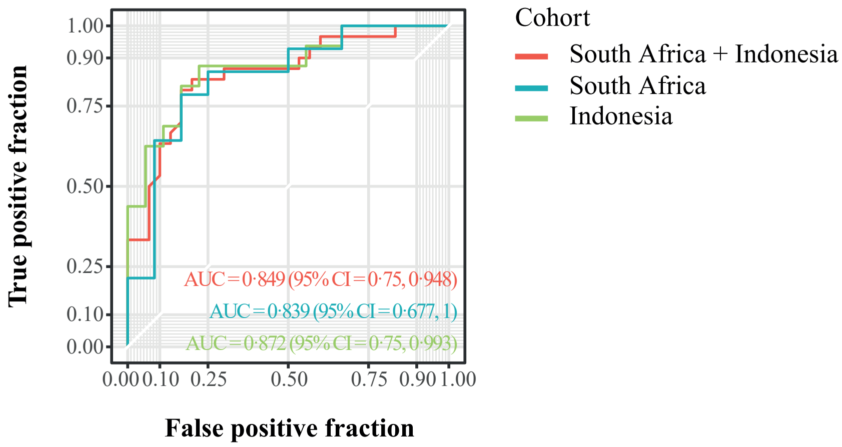

Supplementary Figure S15. Identification of a delta (week two minus diagnosis) signature predicting the outcome of TB treatment. ROC curve showing the predictive power of the gene signature identified in the balanced pooled cohort to classify TB patients into patients who had a good treatment outcome and patients who had a poor treatment outcome, using the RFE - RF model and LOOCV. The dataset was balanced by down-sampling to encompass the same number of individuals with poor and good treatment outcome. Abbreviations: AUC, area under the curve; CI, confidence interval.

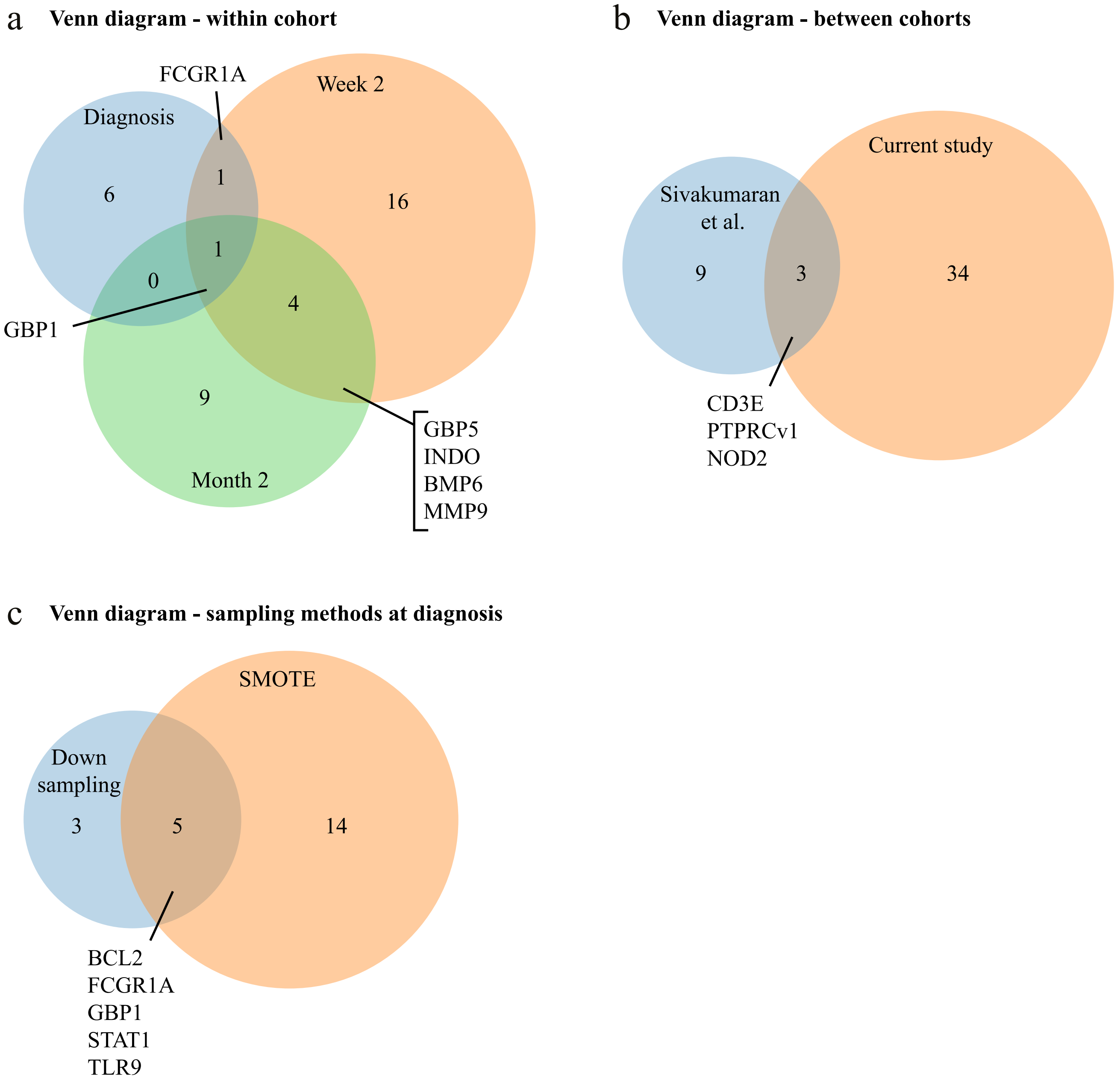

Supplementary Figure S16. Venn diagrams showing the number of genes encompassing treatment outcome signatures as identified by RFE-RF models. (a) Venn diagram displaying the number of unique and overlapping genes comparing diagnosis (blue), week two (orange) and month two (green) gene signatures obtained by random-downsampling. (b) Venn diagram displaying the number of unique and overlapping genes comparing the gene signatures in the current study (diagnosis, week two and month two; orange) obtained by random-downsampling with the gene signatures published by Sivakumaran et al. (blue). (c) Venn diagram displaying the number of unique and overlapping genes comparing the diagnosis gene signature obtained by random-downsampling (blue) with the diagnosis gene signature obtained by applying SMOTE sampling (orange) to balance the classes. Overlapping genes are annotated.

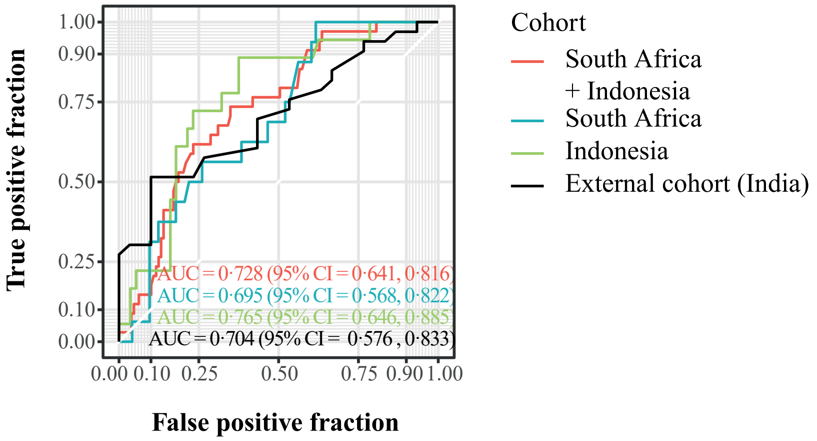

Supplementary Figure S17. Identification of a SMOTE diagnosis signature predicting the outcome of TB treatment. ROC curves showing the predictive power of the gene signatures identified in the balanced pooled cohort (South Africa and Indonesia) and validated in the South African and Indonesian cohort or in an external Indian cohort. TB patients are classified into patients who had a good treatment outcome and patients who had a poor treatment outcome at diagnosis, using the RFE - RF model and LOOCV. The dataset was balanced by SMOTE to encompass the same number of individuals with poor and good treatment outcome. Abbreviations: AUC, area under the curve; CI, confidence interval.
