# Supplementary material for: Transcriptional profiles predict treatment outcome in patients with tuberculosis and diabetes at diagnosis and at two weeks after initiation of anti-tuberculosis treatment
Source: eBioMedicine. 2022 Jul 15;82:104173. doi: 10.1016/j.ebiom.2022.104173 (PMC9297076; doi:10.1016/j.ebiom.2022.104173)
Supplement: Supplementary file 12 [file mmc12.docx]

| First Name | Surname | |
| --- | --- | --- |
| Hazel M. | Dockrell | |
| Jacqueline M. | Cliff | |
| Clare | Eckold | |
| JiSook | Lee | |
| David A. | Moore | |
| Ulla K. | Griffiths | |
| Yoko V. | Laurence | |
| Rob R. | Anmontse | |
| Mihai | Netea | |
| Reinout | van Crevel | |
| Carolien | Ruesen | |
| Ekta | Lachmandas | |
| Stefan H.E. | Kaufmann | |
| Macarena | Beigier | |
| Golinski | Robert | |
| Weiner | January | |
| Simone A. | Joosten | |
| Tom H.M. | Ottenhoff | |
| Frank | Vrieling | |
| Marielle C. | Haks | |
| Gerhard | Walzl | |
| Katharina | Ronacher | |
| Stephanus | Malherbe | |
| Léanie | Kleynhans | |
| Bronwyn | Smith | |
| Kim | Stanley | |
| Gian D. | van der Spuy | |
| André G. | Loxton | |
| Novel N. | Chegou | |
| Marika | Bosman | |
| Leani | Thiart | |
| Chandré | Wagman | |
| Happy | Tshivhula | |
| Mosa | Selamolela | |
| Nicole | Prins | |
| Willem J. | du Plessis | |
| Ilana C. | van Rensburg | |
| Lorinda | du Toit | |
| Julia A. | Critchley | |
| Sarah R. | Kerry | |
| Fiona | Pearson | |
| Daniel | Grint | |
| Mihai | Ioana | |
| Nicolae Mircea | Panduru | |
| Anca L. | Riza | |
| Ramona | Cioboata | |
| Mihaela O. | Dudau | |
| Floarea M. | Nitu | |
| Ileana C. | Bazavan | |
| Mihai | Olteanu | |
| Cornelia D. | Editoiu | |
| Adriana | Florescu | |
| Marius S. | Ciontea | |
| Iulia D. | Capitanescu | |
| Marian | Olaru | |
| Tiberiu | Tataru | |
| Maria D. | Papurica | |
| Ileana | Valutanu | |
| Vasilica | Dubreu | |
| Liviu | Stamatoiu |  |
| Creola | Enoiu |  |
| Maria | Mota |  |
| Simona-  Georgiana | Popa |  |
| Adela G. | Firanescu |  |
| Adina | Popa |  |
| Ioana A. | Gheonea |  |
| Stefania | Bicuti |  |
| Alina | Lepadat |  |
| Ionela  Mihaela | Vladu |  |
| Diana | Clenciu |  |
| Mihaela L. | Bicu |  |
| Costin | Streba |  |
| Alin D. | Demetrian |  |
| Marius | Ciurea |  |
| Alina | Cimpoeru |  |
| Adela | Ciocoiu |  |
| Stefania C. | Dorobantu |  |
| Razvan M. | Plesea |  |
| Elena-  Leocardia | Popescu |  |
| Mihai G. | Cucu |  |
| Ioana | Streata |  |
| Florin | Burada |  |
| Simona | Serban-  Sosoi |  |
| Elena R. | Nicoli |  |
| Susan M. | McAllister |  |
| Philip C. | Hill |  |
| Ajesha E. | Verrall |  |
| Vinod | Kumar |  |
| Cisca | Wijmenga |  |
| Cesar | Ugarte-Gil |  |
| Jorge | Coronel |  |
| Sonia | Lopez |  |
| Ruth | Limascca |  |
| Katherine | Villaizan |  |
| Beatriz | Castro |  |
| Jhomelin | Flores |  |
| Walter | Solano |  |
| Bachti | Alisjahbana |  |
| Rovina | Ruslami |  |
| Nanny N.M. | Soetedjo |  |
| Prayudi | Santoso |  |
| Lidya | Chaidir |  |
| Raspati C. | Koesoemadinata |  |
| Nopi | Susilawati |  |
| Jessi | Annisa |  |
| Resvi | Livia |  |
| Vycke | Yunivita |  |
| Arto Y. | Soeroto |  |
| Hikmat | Permana |  |
| Sofia | Imaculata |  |
| Yuanita | Gunawan |  |
| Nury Fitria | Dewi |  |
| Lika Apriani | Apriani |  |
| Eleonora | Vianello |  |
| Cassandra L.R. | van Doorn |  |
| Suzanne | van Veen |  |
