# Supplementary material for: Transcriptional profiles predict treatment outcome in patients with tuberculosis and diabetes at diagnosis and at two weeks after initiation of anti-tuberculosis treatment
Source: eBioMedicine. 2022 Jul 15;82:104173. doi: 10.1016/j.ebiom.2022.104173 (PMC9297076; doi:10.1016/j.ebiom.2022.104173)
Supplement: Supplementary file 13 [file mmc13.docx]

| First Name | Surname | Affiliation |
| --- | --- | --- |
| Hazel M. | Dockrell | TB Centre and Department of Infection Biology, London School of Hygiene & Tropical Medicine, United Kingdom |
| Jacqueline M. | Cliff | TB Centre and Department of Infection Biology, London School of Hygiene & Tropical Medicine, United Kingdom;  Division of Biosciences, Brunel University London |
| Clare | Eckold | TB Centre and Department of Infection Biology, London School of Hygiene & Tropical Medicine, United Kingdom |
| JiSook | Lee | TB Centre and Department of Infection Biology, London School of Hygiene & Tropical Medicine, United Kingdom |
| David A. | Moore | TB Centre and Department of Clinical Research, London School of Hygiene & Tropical Medicine, United Kingdom |
| Ulla K. | Griffiths | Department of Global Health and Development, London School of Hygiene & Tropical Medicine, United Kingdom |
| Yoko V. | Laurence | TB Centre and Department of Global Health and Development, London School of Hygiene & Tropical Medicine, United Kingdom |
| Rob R. | Anmontse | Radboud university medical center, Dpt Clinical Pharmacy, The Netherlands |
| Mihai | Netea | Radboud university medical center, Dpt Internal Medicine, The Netherlands |
| Reinout | van Crevel | Radboud university medical center, Dpt Internal Medicine, The Netherlands |
| Carolien | Ruesen | Radboud university medical center, Dpt Internal Medicine, The Netherlands |
| Ekta | Lachmandas | Radboud university medical center, Dpt Internal Medicine, The Netherlands |
| Stefan H.E. | Kaufmann | Max Plank Institute for Infection Biology, Berlin, Germany |
| Macarena | Beigier | Max Plank Institute for Infection Biology, Berlin, Germany |
| Golinski | Robert | Max Plank Institute for Infection Biology, Berlin, Germany |
| Weiner | January | Max Plank Institute for Infection Biology, Berlin, Germany |
| Simone A. | Joosten | Infectious Diseases, Leiden University Medical Center, The Netherlands |
| Tom H.M. | Ottenhoff | Infectious Diseases, Leiden University Medical Center, The Netherlands |
| Frank | Vrieling | Infectious Diseases, Leiden University Medical Center, The Netherlands |
| Marielle C. | Haks | Infectious Diseases, Leiden University Medical Center, The Netherlands |
| Gerhard | Walzl | SA MRC Centre for TB Research, DST/NRF Centre of Excellence for Biomedical Tuberculosis Research, Faculty of Medicine and Health Sciences, Stellenbosch  University, Cape Town, South Africa |
| Katharina | Ronacher | SA MRC Centre for TB Research, DST/NRF Centre of Excellence for Biomedical Tuberculosis Research, Faculty of Medicine and Health Sciences, Stellenbosch  University, Cape Town, South Africa; The University of Queensland, Translational Research Institute, Brisbane, Australia |
| Stephanus | Malherbe | SA MRC Centre for TB Research, DST/NRF Centre of Excellence for Biomedical Tuberculosis Research, Faculty of Medicine and Health Sciences, Stellenbosch  University, Cape Town, South Africa |
| Léanie | Kleynhans | SA MRC Centre for TB Research, DST/NRF Centre of Excellence for Biomedical Tuberculosis Research, Faculty of Medicine and Health Sciences, Stellenbosch  University, Cape Town, South Africa |
| Bronwyn | Smith | SA MRC Centre for TB Research, DST/NRF Centre of Excellence for Biomedical Tuberculosis Research, Faculty of Medicine and Health Sciences, Stellenbosch  University, Cape Town, South Africa |
| Kim | Stanley | SA MRC Centre for TB Research, DST/NRF Centre of Excellence for Biomedical Tuberculosis Research, Faculty of Medicine and Health Sciences, Stellenbosch  University, Cape Town, South Africa |
| Gian D. | van der Spuy | SA MRC Centre for TB Research, DST/NRF Centre of Excellence for Biomedical Tuberculosis Research, Faculty of Medicine and Health Sciences, Stellenbosch  University, Cape Town, South Africa |
| André G. | Loxton | SA MRC Centre for TB Research, DST/NRF Centre of Excellence for Biomedical Tuberculosis Research, Faculty of Medicine and Health Sciences, Stellenbosch  University, Cape Town, South Africa |
| Novel N. | Chegou | SA MRC Centre for TB Research, DST/NRF Centre of Excellence for Biomedical Tuberculosis Research, Faculty of Medicine and Health Sciences, Stellenbosch  University, Cape Town, South Africa |
| Marika | Bosman | SA MRC Centre for TB Research, DST/NRF Centre of Excellence for Biomedical Tuberculosis Research, Faculty of Medicine and Health Sciences, Stellenbosch  University, Cape Town, South Africa |
| Leani | Thiart | SA MRC Centre for TB Research, DST/NRF Centre of Excellence for Biomedical Tuberculosis Research, Faculty of Medicine and Health Sciences, Stellenbosch  University, Cape Town, South Africa |
| Chandré | Wagman | SA MRC Centre for TB Research, DST/NRF Centre of Excellence for Biomedical Tuberculosis Research, Faculty of Medicine and Health Sciences, Stellenbosch  University, Cape Town, South Africa |
| Happy | Tshivhula | SA MRC Centre for TB Research, DST/NRF Centre of Excellence for Biomedical Tuberculosis Research, Faculty of Medicine and Health Sciences, Stellenbosch  University, Cape Town, South Africa |
| Mosa | Selamolela | SA MRC Centre for TB Research, DST/NRF Centre of Excellence for Biomedical Tuberculosis Research, Faculty of Medicine and Health Sciences, Stellenbosch  University, Cape Town, South Africa |
| Nicole | Prins | SA MRC Centre for TB Research, DST/NRF Centre of Excellence for Biomedical Tuberculosis Research, Faculty of Medicine and Health Sciences, Stellenbosch  University, Cape Town, South Africa |
| Willem J. | du Plessis | SA MRC Centre for TB Research, DST/NRF Centre of Excellence for Biomedical Tuberculosis Research, Faculty of Medicine and Health Sciences, Stellenbosch  University, Cape Town, South Africa |
| Ilana C. | van Rensburg | SA MRC Centre for TB Research, DST/NRF Centre of Excellence for Biomedical Tuberculosis Research, Faculty of Medicine and Health Sciences, Stellenbosch  University, Cape Town, South Africa |
| Lorinda | du Toit | SA MRC Centre for TB Research, DST/NRF Centre of Excellence for Biomedical Tuberculosis Research, Faculty of Medicine and Health Sciences, Stellenbosch  University, Cape Town, South Africa |
| Julia A. | Critchley | Population Health Research Institute, St George's, University of London, United Kingdom |
| Sarah R. | Kerry | Population Health Research Institute, St George's, University of London, United Kingdom |
| Fiona | Pearson | Population Health Research Institute, St George's, University of London, United Kingdom |
| Daniel | Grint | Population Health Research Institute, St George's, University of London, United Kingdom |
| Mihai | Ioana | Human Genomics Laboratory, Universitatea de Medicină si Farmacie din Craiova, Romania; Dolj Regional Centre of Medical Genetics, Spitalul Clinic Județean  de Urgență Craiova, Romania |
| Mircea  Nicolae | Panduru | Human Genomics Laboratory, Universitatea de Medicina si Farmacie din Craiova, Romania |
| Anca L. | Riza | Human Genomics Laboratory, Universitatea de Medicină și Farmacie din Craiova, Romania; Department of Internal Medicine and Radboud Center for  Infectious Diseases, Radboud University Medical Center, Nijmegen, The Netherlands |
| Ramona | Cioboata | Internal Medicine - Pulmunology Dept., Universitatea de Medicină și Farmacie din Craiova, Romania; Pneumophtisiology Clinic, Spitalul Clinic de Boli  Infecțioase și Pneumoftiziologie "Victor Babeș" Craiova, Romania |
| Mihaela O. | Dudau | Pneumophtisiology Clinic, Spitalul Clinic de Boli Infecțioase și Pneumoftiziologie "Victor Babeș" Craiova, Romania |
| Floarea M. | Nitu | Internal Medicine - Phtisiology Dept., Universitatea de Medicină și Farmacie din Craiova, Romania; Pneumophtisiology Clinic, Spitalul Clinic de Boli Infecțioase  și Pneumoftiziologie "Victor Babeș" Craiova, Romania |
| Ileana C. | Bazavan | Internal Medicine - Phtisiology Dept., Universitatea de Medicină și Farmacie din Craiova, Romania; Pneumophtisiology Clinic, Spitalul Clinic de Boli Infecțioase  și Pneumoftiziologie "Victor Babeș" Craiova, Romania |
| Mihai | Olteanu | Internal Medicine - Phtisiology Dept., Universitatea de Medicină și Farmacie din Craiova, Romania; Pneumophtisiology Clinic, Spitalul Clinic de Boli Infecțioase  și Pneumoftiziologie "Victor Babeș" Craiova, Romania |
| Cornelia D. | Editoiu | TB laboratory, Spitalul Clinic de Boli Infecțioase și Pneumoftiziologie "Victor Babeș" Craiova, Romania |
| Adriana | Florescu | TB laboratory, Spitalul Clinic de Boli Infecțioase și Pneumoftiziologie "Victor Babeș" Craiova, Romania |
| Marius S. | Ciontea | Pulmunology Clinic, Spitalul de Pneumoftiziologie ”Tudor Vladimirescu”, com. Runcu, Gorj, Romania |
| Iulia D. | Capitanescu | Pulmunology Clinic, Spitalul de Pneumoftiziologie ”Tudor Vladimirescu”, com. Runcu, Gorj, Romania |
| Marian | Olaru | Pulmunology Clinic, Spitalul de Pneumoftiziologie ”Tudor Vladimirescu”, com. Runcu, Gorj, Romania |
| Tiberiu | Tataru | Pulmunology Clinic, Spitalul de Pneumoftiziologie ”Tudor Vladimirescu”, com. Runcu, Gorj, Romania |
| Maria D. | Papurica | TB laboratory, Spitalul de Pneumoftiziologie ”Tudor Vladimirescu”, com. Runcu, Gorj, Romania |
| Ileana | Valutanu | TB laboratory, Spitalul de Pneumoftiziologie ”Tudor Vladimirescu”, com. Runcu, Gorj, Romania |
| Vasilica | Dubreu | TB laboratory, Spitalul de Pneumoftiziologie ”Tudor Vladimirescu”, com. Runcu, Gorj, Romania |

| Liviu | Stamatoiu | TB laboratory, Spitalul de Pneumoftiziologie ”Tudor Vladimirescu”, com. Runcu, Gorj, Romania |
| --- | --- | --- |
| Creola | Enoiu | TB laboratory, Spitalul Județean de Urgență Targu-Jiu, Romania |
| Maria | Mota | Nutition and Metabolic Disorders Dept, Universitatea de Medicină și Farmacie din Craiova, Romania; Diabetes, nutrition and metabolic disorders clinic,  Spitalul Clinic Județean de Urgență Craiova, Romania |
| Simona-  Georgiana | Popa | Nutition and Metabolic Disorders Dept, Universitatea de Medicină și Farmacie din Craiova, Romania; Diabetes, nutrition and metabolic disorders clinic,  Spitalul Clinic Județean de Urgență Craiova, Romania |
| Adela G. | Firanescu | Diabetes, Nutrition and Metabolic Disorders Clinic, Spitalul Clinic Județean de Urgență Craiova, Romania |
| Adina | Popa | Diabetes, Nutrition and Metabolic Disorders Clinic, Spitalul Clinic Județean de Urgență Craiova, Romania |
| Ioana A. | Gheonea | Radiology and Imaging Dept., Universitatea de Medicină și Farmacie din Craiova, Romania; Radiology and Medical Imaging Laboratory, Spitalul Clinic  Județean de Urgență Craiova, Romania |
| Stefania | Bicuti | Radiology and Medical Imaging Laboratory, Spitalul Clinic Județean de Urgență Craiova, Romania |
| Alina | Lepadat | Radiology and Medical Imaging Laboratory, Spitalul Clinic Județean de Urgență Craiova, Romania |
| Ionela  Mihaela | Vladu | Nutition and Metabolic Disorders Dept, Universitatea de Medicină și Farmacie din Craiova, Romania; Diabetes, Nutrition and Metabolic Disorders Comp.,  Internal Medicine Dept., Spitalul Clinic Municipal Filantropia Craiova, Romania |
| Diana | Clenciu | Diabetes, Nutrition and Metabolic Disorders Comp., Internal Medicine Dept., Spitalul Clinic Municipal Filantropia Craiova, Romania |
| Mihaela L. | Bicu | Diabetes, Nutrition and Metabolic Disorders Comp., Internal Medicine Dept., Spitalul Clinic Municipal Filantropia Craiova, Romania |
| Costin | Streba | Internal Medicine - Pulmunology Dept., Universitatea de Medicină și Farmacie din Craiova, Romania |
| Alin D. | Demetrian | Thoracic Surgery Dept., Universitatea de Medicină și Farmacie din Craiova, Romania; Thoracic Surgery Clinic, Spitalul Clinic Județean de Urgență Craiova,  Romania |
| Marius | Ciurea | Thoracic Surgery Dept., Universitatea de Medicină și Farmacie din Craiova, Romania; Thoracic Surgery Clinic, Spitalul Clinic Județean de Urgență Craiova,  Romania |
| Alina | Cimpoeru | Human Genomics Laboratory, Universitatea de Medicină si Farmacie din Craiova, Romania |
| Adela | Ciocoiu | Human Genomics Laboratory, Universitatea de Medicină si Farmacie din Craiova, Romania; Dolj Regional Centre of Medical Genetics, Spitalul Clinic Județean  de Urgență Craiova, Romania |
| Stefania C. | Dorobantu | Human Genomics Laboratory, Universitatea de Medicină si Farmacie din Craiova, Romania |
| Razvan M. | Plesea | Human Genomics Laboratory, Universitatea de Medicină si Farmacie din Craiova, Romania; Dolj Regional Centre of Medical Genetics, Spitalul Clinic Județean  de Urgență Craiova, Romania |
| Elena-  Leocardia | Popescu | Human Genomics Laboratory, Universitatea de Medicină si Farmacie din Craiova, Romania; Dolj Regional Centre of Medical Genetics, Spitalul Clinic Județean  de Urgență Craiova, Romania |
| Mihai G. | Cucu | Human Genomics Laboratory, Universitatea de Medicină si Farmacie din Craiova, Romania; Dolj Regional Centre of Medical Genetics, Spitalul Clinic Județean  de Urgență Craiova, Romania |
| Ioana | Streata | Human Genomics Laboratory, Universitatea de Medicină si Farmacie din Craiova, Romania; Dolj Regional Centre of Medical Genetics, Spitalul Clinic Județean  de Urgență Craiova, Romania |
| Florin | Burada | Human Genomics Laboratory, Universitatea de Medicină si Farmacie din Craiova, Romania; Dolj Regional Centre of Medical Genetics, Spitalul Clinic Județean  de Urgență Craiova, Romania |
| Simona | Serban-  Sosoi | Human Genomics Laboratory, Universitatea de Medicină si Farmacie din Craiova, Romania |
| Elena R. | Nicoli | Human Genomics Laboratory, Universitatea de Medicină si Farmacie din Craiova, Romania |
| Susan M. | McAllister | Centre for International Health, University of Otago, New Zealand |
| Philip C. | Hill | Centre for International Health, University of Otago, New Zealand |
| Ajesha E. | Verrall | Centre for International Health, University of Otago, New Zealand |
| Vinod | Kumar | Department of Genetics, University of Groningen, The Netherlands |
| Cisca | Wijmenga | Department of Genetics, University of Groningen, The Netherlands |
| Cesar | Ugarte-Gil | Facultad de Medicina Alberto Hurtado, Universidad Peruana Cayetano Heredia, Lima, Perú |
| Jorge | Coronel | Laboratorio de Investigación de Enfermedades Infecciosas, Universidad Peruana Cayetano Heredia, Lima, Perú |
| Sonia | Lopez | Laboratorio de Investigación de Enfermedades Infecciosas, Universidad Peruana Cayetano Heredia, Lima, Perú |
| Ruth | Limascca | Laboratorio de Investigación de Enfermedades Infecciosas, Universidad Peruana Cayetano Heredia, Lima, Perú |
| Katherine | Villaizan | Laboratorio de Investigación de Enfermedades Infecciosas, Universidad Peruana Cayetano Heredia, Lima, Perú |
| Beatriz | Castro | Laboratorio de Investigación de Enfermedades Infecciosas, Universidad Peruana Cayetano Heredia, Lima, Perú |
| Jhomelin | Flores | Laboratorio de Investigación de Enfermedades Infecciosas, Universidad Peruana Cayetano Heredia, Lima, Perú |
| Walter | Solano | Laboratorio de Investigación de Enfermedades Infecciosas, Universidad Peruana Cayetano Heredia, Lima, Perú |
| Bachti | Alisjahbana | Internal Medicine, Infectious Disease, Universitas Padjadjaran, Bandung, Indonesia |
| Rovina | Ruslami | Pharmacology and Therapy, Universitas Padjadjaran, Bandung, Indonesia |
| Nanny N.M. | Soetedjo | Internal Medicine, Endocrinology, Universitas Padjadjaran, Bandung, Indonesia |
| Prayudi | Santoso | Internal Medicine, Pulmonology, Universitas Padjadjaran, Bandung, Indonesia |
| Lidya | Chaidir | TB-HIV Research Center, Universitas Padjadjaran, Bandung, Indonesia |
| Raspati C. | Koesoemadinata | Microbiology and Parasitology, Universitas Padjadjaran, Bandung, Indonesia |
| Nopi | Susilawati | TB-HIV Research Center, Universitas Padjadjaran, Bandung, Indonesia |
| Jessi | Annisa | TB-HIV Research Center, Universitas Padjadjaran, Bandung, Indonesia |
| Resvi | Livia | TB-HIV Research Center, Universitas Padjadjaran, Bandung, Indonesia |
| Vycke | Yunivita | Pharmacology and Therapy, Universitas Padjadjaran, Bandung, Indonesia |
| Arto Y. | Soeroto | Internal Medicine, Pulmonology, Universitas Padjadjaran, Bandung, Indonesia |
| Hikmat | Permana | Internal Medicine, Endocrinology, Universitas Padjadjaran, Bandung, Indonesia |
| Sofia | Imaculata | TB-HIV Research Center, Universitas Padjadjaran, Bandung, Indonesia |
| Yuanita | Gunawan | TB-HIV Research Center, Universitas Padjadjaran, Bandung, Indonesia |
| Nury Fitria | Dewi | TB-HIV Research Center, Universitas Padjadjaran, Bandung, Indonesia |
| Lika Apriani | Apriani | Public Health, Universitas Padjadjaran, Bandung, Indonesia |
| Eleonora | Vianello | Infectious Diseases, Leiden University Medical Center, The Netherlands |
| Cassandra L.R. | van Doorn | Infectious Diseases, Leiden University Medical Center, The Netherlands |
| Suzanne | van Veen | Infectious Diseases, Leiden University Medical Center, The Netherlands |
